# Effect of nucleos(t)ide analogue discontinuation on the prognosis of HBeAg‐negative hepatitis B virus‐related hepatocellular carcinoma after hepatectomy: A propensity score matching analysis

**DOI:** 10.1002/cam4.70185

**Published:** 2024-09-01

**Authors:** Ting Sun, Yiwen Qiu, Tao Wang, Yi Yang, Haizhou Qiu, Shu Shen, Huasheng Pang, Wentao Wang

**Affiliations:** ^1^ Division of Liver Surgery, Department of General Surgery West China Hospital, Sichuan University Chengdu P. R. China; ^2^ Tibet Center of Disease Control and Prevention Tibet Autonomous Region Lhasa P. R. China

**Keywords:** discontinuation, hepatitis B virus, hepatitis B virus surface antigen, hepatocellular carcinoma, nucleos(t)ide analogues, treatment outcome

## Abstract

**Background:**

Although nucleos(t)ide analogues (NAs) are thought to reduce the risk of hepatitis B virus (HBV)‐related hepatocellular carcinoma (HCC), the effect of NA discontinuation on the prognosis of HBV‐related HCC after hepatectomy is rarely reported. We aimed to investigate the potential for hepatitis B virus e antigen (HBeAg)‐negative HBV‐related HCC patients to discontinue NAs based on preoperative hepatitis B virus surface antigen (HBsAg) status.

**Methods:**

This historical cohort study involved 1232 NA‐treated HBeAg‐negative patients who underwent curative hepatectomy for HBV‐related HCC from 2014 to 2019. The recurrence‐free survival (RFS) and overall survival (OS) of patients discontinuing NAs before surgery were compared with those continuing NAs. Propensity score matching (PSM) was used to balance baseline characteristics.

**Results:**

Of all enrolled patients, 839 (68.1%) patients continued NAs, and 393 (31.9%) patients discontinued NAs. Continuation of NAs was identified as an independent risk factor for RFS (HR 2.047, 95% CI 1.348–3.109, *p* < 0.001 before PSM and HR 2.756, 95% CI 1.537–4.942, *p* < 0.001 after PSM) in HBsAg‐negative patients. Similarly, subgroup survival analyses showed that NA discontinuation was associated with better RFS (*p* = 0.029 before PSM and *p* < 0.001 after PSM) and comparable OS (*p* = 0.935 before PSM and *p* = 0.115 after PSM) than NA continuation in HBsAg‐negative patients. The interaction between HBsAg status and continuation or discontinuation of NAs was significant (*p* for interaction <0.001).

**Conclusions:**

These findings demonstrate the potential for HBeAg‐negative HBV‐related HCC patients who have achieved HBsAg seroclearance to discontinue NAs under strict monitoring.

## 1 INTRODUCTION

Hepatocellular carcinoma (HCC) accounts for approximately 75%–85% of primary liver cancer, which was the third leading cause of cancer death worldwide in 2020, with approximately 830,000 deaths.^1^ Patients with HCC have a dismal prognosis, with median overall survival (OS) ranging from 3 to 60 months.^2^ Chronic infection with hepatitis B virus (HBV) or chronic hepatitis B (CHB) is the main risk factor for HCC and the top contributor to HCC in Asia.^3^ In China, approximately 23 million patients were infected with HBV, and the number of HBV‐associated deaths was nearly 162,000 in 2019.^4^ Because HBV infection accounts for the majority of liver cancer deaths worldwide, control of HBV is one of the major strategies for the treatment of HCC.^5^

Chronic infection with HBV is a dynamic process and tends to present hepatitis B virus e antigen (HBeAg) seroclearance followed by hepatitis B virus surface antigen (HBsAg) seroclearance when patients are undergoing antiviral therapy. It is generally believed that the loss of HBsAg, also called “functional cure,” is the optimal treatment endpoint in CHB patients.^3^ Several studies and guidelines also stated that HBsAg seroclearance is associated with a reduced risk of HCC development.^6^, ^7^ Currently, nucleos(t)ide analogues (NAs), including entecavir (ETV), tenofovir disoproxil fumarate (TDF), etc., are recommended as the first‐line antiviral treatment for CHB in clinical practice due to their low drug resistance.^3^, ^8^, ^9^ However, the uniform criteria for the discontinuation of NAs are still less well established and need to be further improved because of insufficient evidence and limitations of the clinical circumstances. For example, the American Association for the Study of Liver Diseases (AASLD) suggested indefinite antiviral therapy for HBeAg‐negative, immune‐active CHB patients, but the European Association for the Study of the Liver (EASL) recommended that NAs should be discontinued after HBsAg loss.^3^, ^8^ Nevertheless, some clinicians may still recommend the continuation of NAs to prevent relapse for CHB patients regardless of their serological assay results.

It is normally assumed that the prognosis of HCC is primarily associated with the biological behavior of cancer cells and tumor stage, including vascular invasion, satellite nodules, tumor biomarkers, multiple tumor nodules, and tumor size.^10^, ^11^ Moreover, a growing number of studies have indicated that serological indicators may be effective predictors for the prognosis of HBV‐related HCC patients. On the one hand, previous studies have found that positive serum HBeAg was associated with recurrence and poor survival in HBV‐related HCC patients undergoing liver resection.^12^, ^13^ On the other hand, some researchers have also reported the impact of HBsAg seroclearance or preoperative HBsAg levels on the recurrence of HBV‐related HCC after curative resection.^14^, ^15^, ^16^ Considering that NAs are widely used for CHB treatment and contribute to HBeAg/HBsAg seroclearance, NAs are usually essential for HBV‐related HCC patients. However, there is a lack of research regarding the effect of NA discontinuation on the prognosis of HBeAg‐negative HBV‐related HCC and the discontinuation criteria of NAs in HBeAg‐negative patients with HBV‐related HCC.

In this historical cohort study, we aimed to assess the effect of NA discontinuation before liver resection on recurrence and overall survival of HBeAg‐negative HBV‐related HCC patients undergoing liver resection and tried to investigate the potential for them to discontinue NAs based on preoperative serum HBsAg status.

## 2 MATERIALS AND METHODS

### 2.1 Patients

The present historical cohort study was conducted on consecutive HBeAg‐negative HBV‐related HCC patients who underwent curative liver resection as first‐line therapy at West China Hospital, Sichuan University, from January 2014 to December 2019.

The inclusion criteria were as follows: (1) patients who received R0 liver resection as an initial treatment and had not received any other antitumor therapies before surgery; (2) patients with a histopathological diagnosis of HCC; and (3) patients who (1) underwent continuous antiviral treatment with NAs before surgery for at least 3 years and consistently continued NAs for the entire follow‐up period (continuation of NAs group) or (2) had previously undergone antiviral treatment with NAs for at least 3 years but had discontinued NAs before surgery for at least 1 year and were in the NA‐withdrawal state for the entire follow‐up period (discontinuation of NAs group). Continuation or discontinuation of NAs was performed and the period of discontinuation mentioned above was set according to the EASL guidelines^3^ or the Asian Pacific Association for the Study of the Liver (APASL) guidelines.^9^, ^17^ Specifically, treatment could be stopped (1) after HBsAg loss, with or without anti‐HBs seroconversion, (2) after at least 1 year of additional therapy after HBeAg seroconversion with undetectable HBV DNA, or (3) after treatment for at least 2 years with undetectable HBV DNA. The stopping of NA treatment could be considered in cirrhotic patients under strict monitoring. After stopping of NAs, patients should be monitored monthly for the initial 3 months and then every 3–6 months.

The exclusion criteria were as follows: (1) positive HBeAg status in preoperative assessments; (2) coinfection with hepatitis C virus; (3) extrahepatic metastasis; (4) missing data or loss to follow‐up; (5) treatment with antiviral drugs for HBV other than NAs (e.g., interferon); and (6) virological relapse or HBV reactivation before surgery.

### 2.2 Perioperative data

All relevant demographic characteristics and medication use were ascertained from self‐reports and medical record abstraction. Alcohol consumption and cigarette smoking were categorized on the basis of current or past use. Diabetes mellitus/hypertension was defined as a diabetes/hypertension diagnosis by a physician or other health professional. Routine preoperative assessments consisted of serological tests and imaging examinations. Hepatitis B serology, HBV DNA load, serum alphafetoprotein (AFP), liver function test, etc., were included. All patients underwent preoperative contrast‐enhanced computed tomography (CT) and/or magnetic resonance imaging (MRI), and tumor resectability was carefully evaluated by experienced surgeons.

Intraoperative data, including physical status, blood loss, operation time, blood transfusion and hepatectomy method (anatomical liver resection or nonanatomic liver resection), were recorded. Physical status was evaluated according to the American Society of Anesthesiologists (ASA) grading system. Anatomical liver resection was defined by the Brisbane 2000 nomenclature of liver anatomy.^18^

Professional pathologists examined all resected specimens and characterized pathologic findings, including tumor number and size, the presence of microvascular invasion and cirrhosis, tumor differentiation, etc.

### 2.3 Follow‐up and study outcomes

All patients were followed up once every 2 months for the first 2 years after surgery and once every 3 months thereafter until death or dropout from follow‐up. Contrast‐enhanced CT, MRI and serum AFP were included in follow‐up examinations and were mainly used to diagnose tumor recurrence. Treatment choices for patients diagnosed with tumor recurrence included repeat liver resection, transarterial chemoembolization, radiofrequency ablation, systemic therapy, liver transplantation or supportive treatment based on general conditions of patients and patterns of tumor recurrence. The primary endpoint was recurrence‐free survival (RFS) and the secondary endpoint was OS. RFS was defined as the period from surgery to first recurrence. OS was defined as the interval time between surgery and death.

### 2.4 Statistical analysis

Parametric continuous data are summarized as the mean ± standard deviation (SD), and nonparametric continuous data are summarized as the median and interquartile range (Q1–Q3). Categorical data are summarized as frequencies and percentages. Statistical comparison of continuous variables was performed using Student's *t*‐test or the Mann–Whitney *U* test as appropriate. Statistical comparison of categorical variables was performed using the Chi‐square test or Fisher's exact test as appropriate. Survival analyses were performed using the Kaplan–Meier method, and the log‐rank test was used for comparison. The Cox proportional hazards regression model was utilized to identify independent prognostic factors of OS and RFS. Propensity score matching (PSM) was used to balance important baseline characteristics between all patients or HBsAg‐negative patients continuing NAs and discontinuing NAs. A 1:1 matching on the propensity score was performed using the nearest‐neighbor matching algorithm with a caliper width equal to 0.01 (for all patients) or 0.1 (for HBsAg‐negative patients) of the SD of the logit of the propensity score. Interactions of subgroups were evaluated by the likelihood ratio test. All the reported *p*‐values are two‐tailed, and *p* < 0.05 was considered statistically significant. All statistical analyses were performed using Empower® (www.empowerstats.com, X&Y Solutions Inc., Boston MA).

## 3 RESULTS

### 3.1 Baseline characteristics

**FIGURE 1:**
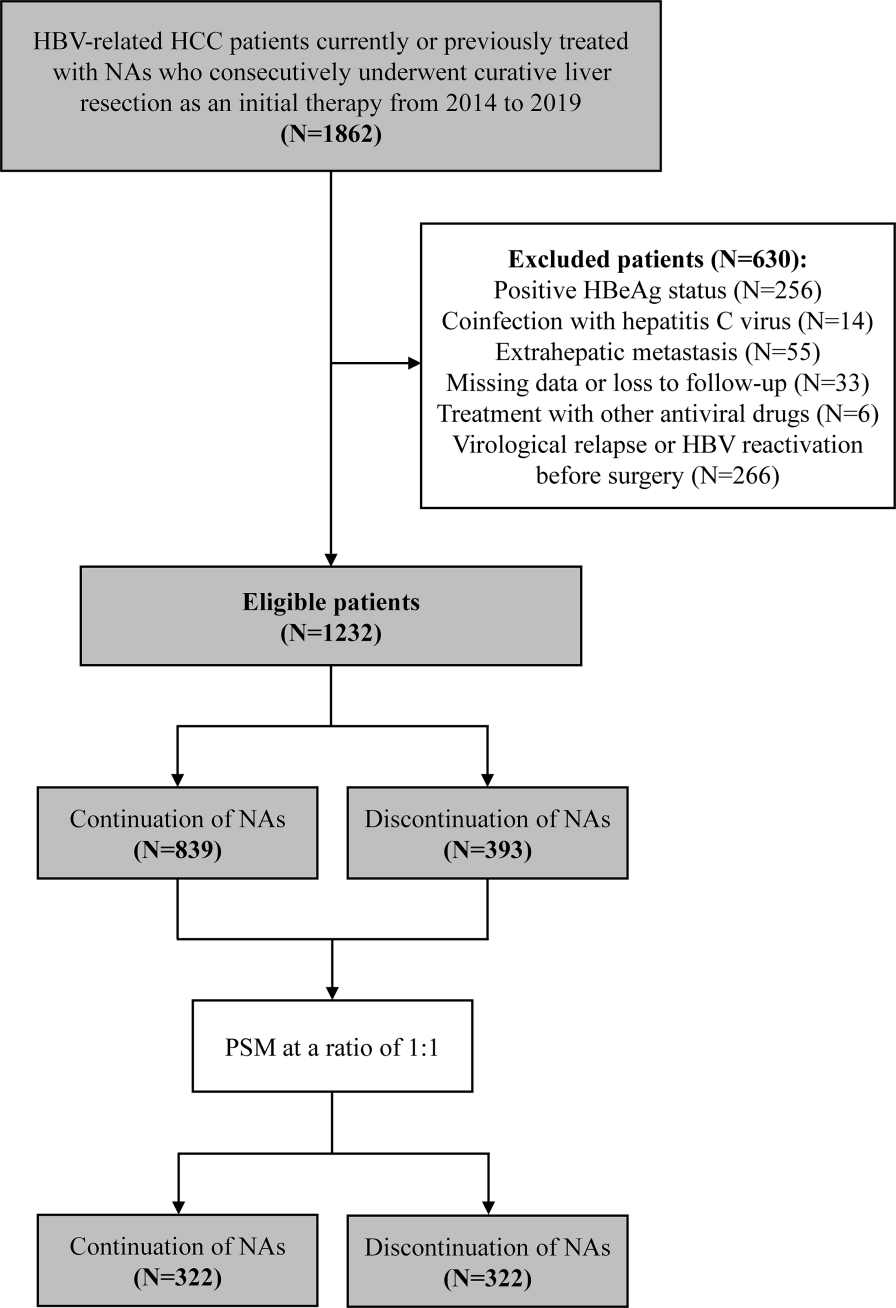
Flow chart of patient selection and propensity score matching (PSM) process.

According to the inclusion and exclusion criteria, we enrolled 1232 eligible patients, of whom 839 (68.1%) patients continued NA therapy and 393 (31.9%) patients discontinued NA therapy before surgery (Figure 1). The median follow‐up time for all patients was 34.7 months (interquartile range, 22.3–50.5 months). As shown in Table 1, compared with patients in the discontinuation of NAs group, patients in the continuation of NAs group had more instances of preoperative positive HBsAg status (87.6% vs. 61.8%, *p* < 0.001), fewer instances of positive HBsAb status (14.4% vs. 29.8%, *p* < 0.001) and a higher percentage of HBV DNA > 10^3^ IU/mL (44.6% vs. 37.2%, *p* = 0.014). Some other baseline characteristics of the patients were also significantly different between the two groups before PSM. After PSM, 322 pairs of patients who continued and discontinued NAs were matched, and there were no significant differences between the two groups (Table 1).

**TABLE 1:** Baseline characteristics of all hepatocellular carcinoma (HCC) patients before and after propensity score matching (PSM).

Variables	Before PSM			After PSM		
	Continuation of NAs (*N* = 839)	Discontinuation of NAs (*N* = 393)	*p*‐value	Continuation of NAs (*N* = 322)	Discontinuation of NAs (*N* = 322)	*p*‐value
*Demographic characteristics*						
Age, years	53.5 ± 11.1	55.1 ± 12.0	**0.032**	54.5 ± 12.0	54.9 ± 12.1	0.598
Male sex	721 (85.9%)	345 (87.8%)	0.375	280 (87.0%)	279 (86.6%)	0.907
BMI, kg/m^2^	23.1 ± 3.1	23.2 ± 3.3	0.670	23.2 ± 3.2	23.1 ± 3.2	0.641
Alcohol consumption	330 (39.3%)	170 (43.3%)	0.191	138 (42.9%)	134 (41.6%)	0.750
Cigarette smoking	434 (51.7%)	221 (56.2%)	0.140	171 (53.1%)	177 (55.0%)	0.635
Diabetes mellitus	62 (7.4%)	37 (9.4%)	0.223	28 (8.7%)	33 (10.2%)	0.501
Hypertension	150 (17.9%)	66 (16.8%)	0.641	68 (21.1%)	57 (17.7%)	0.273
Type of NA						
ETV	668 (79.6%)	316 (80.4%)	0.915	256 (79.5%)	253 (78.6%)	0.941
TDF	39 (4.6%)	16 (4.1%)		12 (3.7%)	15 (4.7%)	
Other	32 (3.8%)	17 (4.3%)		14 (4.3%)	15 (4.7%)	
Exposure to two or more types of NAs	100 (11.9%)	44 (11.2%)		40 (12.4%)	39 (12.1%)	
*Laboratory findings*						
HBsAg‐positive	735 (87.6%)	243 (61.8%)	**<0.001**	243 (75.5%)	234 (72.7%)	0.418
HBsAb‐positive	121 (14.4%)	117 (29.8%)	**<0.001**	72 (22.4%)	78 (24.2%)	0.576
HBeAb‐positive	750 (89.4%)	337 (85.8%)	0.065	272 (84.5%)	285 (88.5%)	0.134
HBV DNA, IU/mL						
≤10^3^	465 (55.4%)	247 (62.8%)	**0.014**	183 (56.8%)	187 (58.1%)	0.750
>10^3^	374 (44.6%)	146 (37.2%)		139 (43.2%)	135 (41.9%)	
AFP, ng/mL						
≤400	540 (64.4%)	221 (56.2%)	**0.006**	194 (60.2%)	189 (58.7%)	0.688
>400	299 (35.6%)	172 (43.8%)		128 (39.8%)	133 (41.3%)	
Hemoglobin, g/L	144.0 [133.0–155.0]	143.0 [132.0–153.0]	0.355	144.0 [132.0–155.0]	143.0 [132.0–153.0]	0.789
Platelets, 10^9^/L	129.0 [94.0–175.5]	150.0 [104.0–200.0]	**<0.001**	148.5 [103.0–197.0]	144.0 [99.0–193.0]	0.458
ALT, IU/L	34.0 [24.0–50.0]	33.0 [21.0–52.0]	0.547	35.0 [24.0–51.8]	32.0 [21.0–49.0]	0.416
AST, IU/L	34.0 [27.0–47.0]	37.0 [27.0–58.0]	**0.004**	38.0 [27.0–52.0]	37.0 [27.0–55.0]	0.991
TBIL, μmol/L	13.4 [10.3–17.7]	13.6 [10.6–18.0]	0.415	13.4 [10.0–18.0]	13.7 [10.6–17.7]	0.549
Albumin, g/L	42.9 [40.3–45.6]	42.1 [39.6–44.8]	**0.003**	42.2 [39.7–45.0]	42.1 [39.4–44.6]	0.893
PT, s	12.0 [11.4–12.7]	12.1 [11.4–12.7]	0.651	12.0 [11.4–12.6]	12.1 [11.4–12.7]	0.259
Child–Pugh grade						
A	831 (99.0%)	389 (99.0%)	0.915	320 (99.4%)	320 (99.4%)	1.000
B	8 (1.0%)	4 (1.0%)		2 (0.6%)	2 (0.6%)	
*Surgical data*						
ASA grade						
I	550 (65.6%)	238 (60.6%)	0.089	194 (60.2%)	196 (60.9%)	0.872
II	289 (34.4%)	155 (39.4%)		128 (39.8%)	126 (39.1%)	
Blood loss, mL	200.0 [100.0–400.0]	300.0 [200.0–500.0]	**<0.001**	300.0 [200.0–400.0]	300.0 [200.0–500.0]	0.796
Operation time, min	200.0 [160.0–250.0]	220.0 [175.0–270.0]	**<0.001**	225.0 [175.0–273.8]	215.0 [175.0–265.0]	0.476
Blood transfusion	52 (6.2%)	27 (6.9%)	0.653	27 (8.4%)	23 (7.1%)	0.556
Anatomic resection	269 (32.1%)	156 (39.7%)	**0.009**	115 (35.7%)	127 (39.4%)	0.329
*Pathologic findings*						
Single tumor	731 (87.1%)	320 (81.4%)	**0.008**	266 (82.6%)	268 (83.2%)	0.834
Tumor size, cm						
≤5	504 (60.1%)	152 (38.7%)	**<0.001**	134 (41.6%)	141 (43.8%)	0.577
>5	335 (39.9%)	241 (61.3%)		188 (58.4%)	181 (56.2%)	
MVI	224 (26.7%)	142 (36.1%)	**<0.001**	110 (34.2%)	107 (33.2%)	0.803
Satellite nodule	78 (9.3%)	44 (11.2%)	0.298	44 (13.7%)	36 (11.2%)	0.339
PVTT	49 (5.8%)	51 (13.0%)	**<0.001**	41 (12.7%)	37 (11.5%)	0.629
Cirrhosis	443 (52.8%)	173 (44.0%)	**0.004**	155 (48.1%)	161 (50.0%)	0.636
Edmondson‐Steiner grade						
≤II	452 (53.9%)	212 (53.9%)	0.982	155 (48.1%)	174 (54.0%)	0.134
≥III	387 (46.1%)	181 (46.1%)		167 (51.9%)	148 (46.0%)	
BCLC stage						
0/A	718 (85.6%)	289 (73.5%)	**<0.001**	242 (75.2%)	245 (76.1%)	0.783
B/C	121 (14.4%)	104 (26.5%)		80 (24.8%)	77 (23.9%)	

*Note*: Bold text indicated that these variables were statistically significant.

Abbreviations: AFP, alpha‐fetoprotein; ALT, alanine aminotransferase; ASA, American Society of Anesthesiologists; AST, aspartate aminotransferase; BCLC, Barcelona Clinic Liver Cancer; BMI, body mass index; ETV, entecavir; HBeAb, hepatitis B e antibody; HBeAg, hepatitis B virus e antigen; HBsAb, hepatitis B surface antibody; HBsAg, hepatitis B surface antigen; HBV, hepatitis B virus; HCC, hepatocellular carcinoma; MVI, microvascular invasion; NAs, nucleos(t)ide analogues; PSM, propensity score matching; PT, prothrombin time; PVTT, portal vein tumor thrombus; TBIL, total bilirubin; TDF, tenofovir disoproxil fumarate.

### 3.2 Univariate and multivariate Cox regression analyses of RFS and OS before and after PSM

Before PSM, univariate and multivariate Cox regression analyses were performed to identify independent prognostic factors for RFS and OS in all HCC patients (Table 2). The results showed that the continuation of NAs (HR 0.623, 95% CI 0.526–0.737, *p* < 0.001) was an independent predictor of RFS. Continuation of NAs (HR 0.243, 95% CI 0.198–0.297, *p* < 0.001), positive HBsAg status (HR 2.279, 95% CI 1.667–3.116, *p* < 0.001), etc., were independent prognostic factors for OS. Moreover, we performed Cox regression analysis of RFS and OS stratified by preoperative HBsAg status before PSM. Notably, we found that the continuation of NAs was an independent risk factor for RFS (HR 2.047, 95% CI 1.348–3.109, *p* < 0.001) and not an independent predictor for OS in HBsAg‐negative HCC patients (Table S1). However, the continuation of NAs was still an independent protective factor for RFS (HR 0.419, 95% CI 0.347–0.507, *p* < 0.001) and OS (HR 0.190, 95% CI 0.153–0.237, *p* < 0.001) in HBsAg‐positive HCC patients (Table S2).

**TABLE 2:** Univariate and multivariate Cox regression analysis of recurrence‐free survival (RFS) and overall survival (OS) in all hepatocellular carcinoma (HCC) patients before propensity score matching (PSM) (*N* = 1232).

Variables	RFS				OS			
	Univariate		Multivariate		Univariate		Multivariate	
	HR (95% CI)	*p*‐value	HR (95% CI)	*p*‐value	HR (95% CI)	*p*‐value	HR (95% CI)	*p*‐value
Age, years	0.991 (0.984–0.997)	**0.007**	0.989 (0.982–0.996)	**0.003**	0.981 (0.973–0.990)	**<0.001**	0.989 (0.979–0.999)	**0.036**
Male sex	1.146 (0.912–1.441)	0.243			1.156 (0.869–1.538)	0.318		
BMI, kg/m^2^	0.993 (0.970–1.017)	0.550			0.976 (0.947–1.005)	0.105		
Alcohol consumption	1.013 (0.868–1.184)	0.867			1.104 (0.912–1.335)	0.310		
Cigarette smoking	1.147 (0.985–1.337)	0.078			1.295 (1.070–1.567)	**0.008**	1.283 (1.054–1.563)	**0.013**
Diabetes mellitus	1.029 (0.773–1.370)	0.844			1.144 (0.820–1.596)	0.429		
Hypertension	0.855 (0.696–1.051)	0.137			0.691 (0.524–0.911)	**0.009**	1.177 (0.822–1.684)	0.373
ETV monotherapy	1.121 (0.923–1.361)	0.248			1.145 (0.896–1.463)	0.278		
NAs, continuation vs. discontinuation	0.563 (0.481–0.659)	**<0.001**	0.623 (0.526–0.737)	**<0.001**	0.287 (0.238–0.347)	**<0.001**	0.243 (0.198–0.297)	**<0.001**
HBsAg‐positive	1.123 (0.927–1.362)	0.235			1.489 (1.151–1.927)	**0.002**	2.279 (1.667–3.116)	**<0.001**
HBsAb‐positive	0.919 (0.753–1.120)	0.402			0.802 (0.622–1.034)	0.088		
HBeAb‐positive	1.173 (0.922–1.491)	0.194			1.663 (1.177–2.349)	**0.004**	0.905 (0.621–1.317)	0.601
HBV DNA, IU/mL, >10^3^ vs. ≤10^3^	1.300 (1.116–1.515)	**<0.001**	1.161 (0.985–1.368)	0.075	1.612 (1.335–1.945)	**<0.001**	1.258 (1.016–1.558)	**0.035**
AFP, ng/mL, >400 vs. ≤400	1.843 (1.581–2.149)	**<0.001**	1.297 (1.097–1.534)	**0.002**	2.457 (2.033–2.970)	**<0.001**	1.562 (1.271–1.920)	**<0.001**
Hemoglobin, g/L	0.992 (0.988–0.997)	**<0.001**	0.996 (0.991–1.000)	0.057	0.995 (0.990–1.001)	0.083		
Platelets, 10^9^/L	1.003 (1.002–1.004)	**<0.001**	1.000 (0.999–1.001)	0.666	1.003 (1.002–1.005)	**<0.001**	0.999 (0.998–1.001)	0.442
ALT, IU/L	1.001 (1.000–1.002)	**0.017**	1.000 (0.997–1.003)	0.967	1.002 (1.001–1.003)	**0.004**	0.998 (0.995–1.002)	0.331
AST, IU/L	1.002 (1.001–1.003)	**<0.001**	1.000 (0.997–1.003)	0.869	1.003 (1.002–1.004)	**<0.001**	1.001 (0.998–1.005)	0.459
TBIL, μmol/L	1.001 (0.990–1.012)	0.865			1.011 (1.005–1.018)	**<0.001**	1.007 (1.001–1.014)	**0.029**
Albumin, g/L	0.941 (0.924–0.958)	**<0.001**	0.970 (0.950–0.989)	**0.002**	0.925 (0.904–0.945)	**<0.001**	0.967 (0.942–0.992)	**0.010**
PT, s	1.053 (0.995–1.114)	0.072			1.114 (1.044–1.189)	**0.001**	1.028 (0.945–1.117)	0.526
Child–Pugh grade, A vs. B	1.459 (0.605–3.519)	0.400			1.059 (0.396–2.835)	0.909		
ASA grade, II vs. I	0.935 (0.797–1.096)	0.407			0.766 (0.625–0.938)	**0.010**	0.933 (0.701–1.240)	0.632
Blood loss, mL	1.000 (1.000–1.000)	**<0.001**	1.000 (1.000–1.000)	0.488	1.000 (1.000–1.000)	**<0.001**	1.000 (1.000–1.000)	0.078
Operation time, min	1.003 (1.002–1.004)	**<0.001**	1.001 (1.000–1.002)	0.100	1.004 (1.003–1.005)	**<0.001**	1.001 (0.999–1.002)	0.274
Blood transfusion	1.620 (1.220–2.150)	**<0.001**	0.894 (0.632–1.266)	0.529	1.869 (1.354–2.579)	**<0.001**	0.929 (0.621–1.389)	0.718
Anatomic resection	1.078 (0.919–1.264)	0.354			1.171 (0.964–1.423)	0.111		
Single tumor	0.549 (0.452–0.666)	**<0.001**	0.916 (0.651–1.288)	0.613	0.560 (0.445–0.706)	**< 0.001**	1.188 (0.765–1.846)	0.442
Tumor size, cm, >5 vs. ≤5	2.745 (2.345–3.213)	**<0.001**	1.859 (1.551–2.229)	**<0.001**	2.952 (2.415–3.609)	**<0.001**	1.618 (1.289–2.030)	**<0.001**
MVI	2.105 (1.797, 2.467)	**<0.001**	1.355 (1.139–1.612)	**<0.001**	2.543 (2.101–3.079)	**<0.001**	1.492 (1.202–1.851)	**<0.001**
Satellite nodule	2.617 (2.114–3.241)	**<0.001**	1.825 (1.458–2.284)	**<0.001**	2.094 (1.626–2.698)	**<0.001**	1.417 (1.079–1.861)	**0.012**
PVTT	2.656 (2.083–3.387)	**<0.001**	0.933 (0.629–1.383)	0.729	3.816 (2.963–4.913)	**<0.001**	0.925 (0.566–1.510)	0.754
Cirrhosis	1.025 (0.880–1.193)	0.754			1.020 (0.845–1.231)	0.838		
Edmondson‐Steiner grade, ≥III vs. ≤II	1.550 (1.330–1.805)	**<0.001**	1.296 (1.101–1.524)	**0.002**	1.963 (1.621–2.376)	**<0.001**	1.557 (1.268–1.911)	**<0.001**
BCLC stage, 0/A vs. B/C	0.376 (0.315–0.448)	**<0.001**	0.609 (0.418–0.889)	**0.010**	0.307 (0.251–0.375)	**<0.001**	0.461 (0.283–0.752)	**0.002**

*Note*: Bold text indicated that these variables were statistically significant.

Abbreviations: AFP, alpha‐fetoprotein; ALT, alanine aminotransferase; ASA, American Society of Anesthesiologists; AST, aspartate aminotransferase; BCLC, Barcelona Clinic Liver Cancer; BMI, body mass index; CI, confidence interval; ETV, entecavir; HBeAb, hepatitis B e antibody; HBsAb, hepatitis B surface antibody; HBsAg, hepatitis B surface antigen; HBV, hepatitis B virus; HCC, hepatocellular carcinoma; HR, hazard ratio; MVI, microvascular invasion; NAs, nucleos(t)ide analogues; OS, overall survival; PSM, propensity score matching; PT, prothrombin time; PVTT, portal vein tumor thrombus; RFS, recurrence‐free survival; TBIL, total bilirubin.

After PSM, the results of the Cox regression analysis (Table 3) identified that the continuation of NAs (HR 0.569, 95% CI 0.463–0.700, *p* < 0.001) was an independent prognostic factor for RFS. Continuation of NAs (HR 0.270, 95% CI 0.211–0.345, *p* < 0.001), positive HBsAg status (HR 1.577, 95% CI 1.014–2.454, *p* = 0.043), etc., were independent predictors for OS. In addition, we also performed Cox regression analysis of RFS and OS stratified by preoperative HBsAg status after PSM. Similarly, the continuation of NAs was identified as an independent risk factor for RFS (HR 2.756, 95% CI 1.537–4.942, *p* < 0.001) and not an independent predictor for OS in HBsAg‐negative HCC patients (Table S3). However, the continuation of NAs was an independent protective factor for RFS (HR 0.400, 95% CI 0.315–0.506, *p* < 0.001) and OS (HR 0.218, 95% CI 0.166–0.287, *p* < 0.001) in HBsAg‐positive HCC patients (Table S4).

**TABLE 3:** Univariate and multivariate Cox regression analysis of recurrence‐free survival (RFS) and overall survival (OS) in all hepatocellular carcinoma (HCC) patients after propensity score matching (PSM) (*N* = 644).

Variables	RFS				OS			
	Univariate		Multivariate		Univariate		Multivariate	
	HR (95% CI)	*p*‐value	HR (95% CI)	*p*‐value	HR (95% CI)	*p*‐value	HR (95% CI)	*p*‐value
Age, years	0.980 (0.972–0.989)	**<0.001**	0.983 (0.973–0.993)	**0.001**	0.973 (0.964–0.982)	**<0.001**	0.986 (0.974–0.998)	**0.027**
Male sex	1.234 (0.909–1.674)	0.177			1.102 (0.788–1.543)	0.570		
BMI, kg/m^2^	0.970 (0.940–1.001)	0.059			0.947 (0.913–0.982)	**0.003**	0.997 (0.959–1.036)	0.868
Alcohol consumption	0.983 (0.805–1.201)	0.867			0.988 (0.787–1.239)	0.913		
Cigarette smoking	1.098 (0.900–1.339)	0.358			1.134 (0.905–1.419)	0.275		
Diabetes mellitus	0.784 (0.546–1.126)	0.187			0.799 (0.531–1.202)	0.281		
Hypertension	0.768 (0.591–0.999)	**0.049**	1.004 (0.749–1.348)	0.976	0.589 (0.425–0.816)	**0.001**	0.913 (0.587–1.421)	0.686
ETV monotherapy	1.184 (0.922–1.521)	0.186			1.176 (0.882–1.568)	0.270		
NAs, continuation vs. discontinuation	0.716 (0.587–0.873)	**<0.001**	0.569 (0.463–0.700)	**<0.001**	0.375 (0.296–0.475)	**<0.001**	0.270 (0.211–0.345)	**<0.001**
HBsAg‐positive	1.507 (1.188–1.913)	**<0.001**	0.909 (0.648–1.277)	0.583	2.760 (1.999–3.810)	**<0.001**	1.577 (1.014–2.454)	**0.043**
HBsAb‐positive	0.684 (0.533–0.876)	**0.003**	0.941 (0.681–1.300)	0.710	0.480 (0.350–0.658)	**<0.001**	0.924 (0.626–1.363)	0.689
HBeAb‐positive	1.293 (0.963–1.736)	0.087			2.232 (1.471–3.387)	**<0.001**	0.898 (0.565–1.427)	0.649
HBV DNA, IU/mL, >10^3^ vs. ≤10^3^	1.412 (1.159–1.721)	**<0.001**	1.059 (0.847–1.324)	0.613	1.951 (1.559–2.441)	**<0.001**	1.210 (0.937–1.564)	0.144
AFP, ng/mL, >400 vs. ≤400	1.957 (1.605–2.386)	**<0.001**	1.395 (1.118–1.740)	**0.003**	2.194 (1.753–2.747)	**<0.001**	1.443 (1.130–1.842)	**0.003**
Hemoglobin, g/L	0.991 (0.985–0.996)	**<0.001**	0.992 (0.986–0.998)	**0.013**	0.994 (0.988–1.001)	0.072		
Platelets, 10^9^/L	1.002 (1.001–1.004)	**<0.001**	1.000 (0.998–1.001)	0.583	1.002 (1.001–1.004)	**<0.001**	1.000 (0.998–1.001)	0.839
ALT, IU/L	1.001 (1.000–1.002)	0.130			1.001 (1.000–1.002)	0.154		
AST, IU/L	1.002 (1.001–1.003)	**0.003**	1.000 (0.998–1.002)	0.824	1.002 (1.001–1.003)	**0.002**	1.000 (0.998–1.002)	0.819
TBIL, μmol/L	0.992 (0.977–1.007)	0.304			1.002 (0.986–1.018)	0.842		
Albumin, g/L	0.956 (0.934–0.978)	**<0.001**	0.977 (0.952–1.003)	0.086	0.939 (0.915–0.963)	**<0.001**	0.965 (0.936–0.993)	**0.016**
PT, s	1.123 (1.020–1.237)	**0.018**	1.005 (0.905–1.117)	0.920	1.283 (1.158–1.423)	**<0.001**	1.088 (0.969–1.222)	0.154
Child–Pugh grade, A vs. B	0.866 (0.278–2.699)	0.805			0.966 (0.240–3.880)	0.961		
ASA grade, II vs. I	0.817 (0.667–1.002)	0.053			0.624 (0.490–0.793)	**<0.001**	1.073 (0.763–1.508)	0.687
Blood loss, mL	1.000 (1.000–1.000)	**<0.001**	1.000 (1.000–1.000)	0.760	1.000 (1.000–1.000)	**<0.001**	1.000 (1.000–1.000)	0.453
Operation time, min	1.002 (1.001–1.004)	**<0.001**	1.001 (0.999–1.002)	0.293	1.003 (1.002–1.005)	**<0.001**	1.001 (1.000, 1.003)	0.172
Blood transfusion	1.537 (1.091–2.166)	**0.014**	1.044 (0.657–1.658)	0.855	1.716 (1.189–2.475)	**0.004**	0.882 (0.520–1.497)	0.642
Anatomic resection	0.932 (0.758–1.145)	0.500			1.080 (0.859–1.359)	0.509		
Single tumor	0.670 (0.525–0.857)	**0.001**	0.892 (0.547–1.454)	0.646	0.633 (0.482–0.830)	**<0.001**	1.104 (0.611–1.997)	0.742
Tumor size, cm, >5 vs. ≤5	2.529 (2.037–3.140)	**<0.001**	1.975 (1.548–2.519)	**<0.001**	2.347 (1.835–3.003)	**<0.001**	1.622 (1.234–2.131)	**<0.001**
MVI	2.091 (1.708–2.559)	**<0.001**	1.442 (1.149–1.809)	**0.002**	2.416 (1.926–3.029)	**<0.001**	1.574 (1.212–2.044)	**<0.001**
Satellite nodule	2.413 (1.856–3.138)	**<0.001**	1.607 (1.199–2.152)	**0.001**	1.717 (1.279–2.305)	**<0.001**	1.212 (0.865–1.698)	0.265
PVTT	2.532 (1.923–3.335)	**<0.001**	0.997 (0.565–1.760)	0.993	2.938 (2.208–3.911)	**<0.001**	0.815 (0.417–1.591)	0.548
Cirrhosis	0.976 (0.801–1.189)	0.806			1.114 (0.891–1.392)	0.345		
Edmondson‐Steiner grade, ≥III vs. ≤II	1.657 (1.358–2.022)	**<0.001**	1.378 (1.112–1.707)	**0.003**	2.118 (1.682–2.666)	**<0.001**	1.822 (1.428–2.325)	**<0.001**
BCLC stage, 0/A vs. B/C	0.420 (0.338–0.520)	**<0.001**	0.626 (0.363–1.079)	0.092	0.350 (0.277–0.442)	**<0.001**	0.421 (0.221–0.802)	**0.009**

*Note*: Bold text indicated that these variables were statistically significant.

Abbreviations: AFP, alpha‐fetoprotein; ALT, alanine aminotransferase; ASA, American Society of Anesthesiologists; AST, aspartate aminotransferase; BCLC, Barcelona Clinic Liver Cancer; BMI, body mass index; CI, confidence interval; ETV, entecavir; HBeAb, hepatitis B e antibody; HBsAb, hepatitis B surface antibody; HBsAg, hepatitis B surface antigen; HBV, hepatitis B virus; HCC, hepatocellular carcinoma; HR, hazard ratio; MVI, microvascular invasion; NAs, nucleos(t)ide analogues; OS, overall survival; PSM, propensity score matching; PT, prothrombin time; PVTT, portal vein tumor thrombus; RFS, recurrence‐free survival; TBIL, total bilirubin.

### 3.3 Overall impact of continuation of NAs or discontinuation of NAs on RFS and OS before and after PSM

**FIGURE 2:**
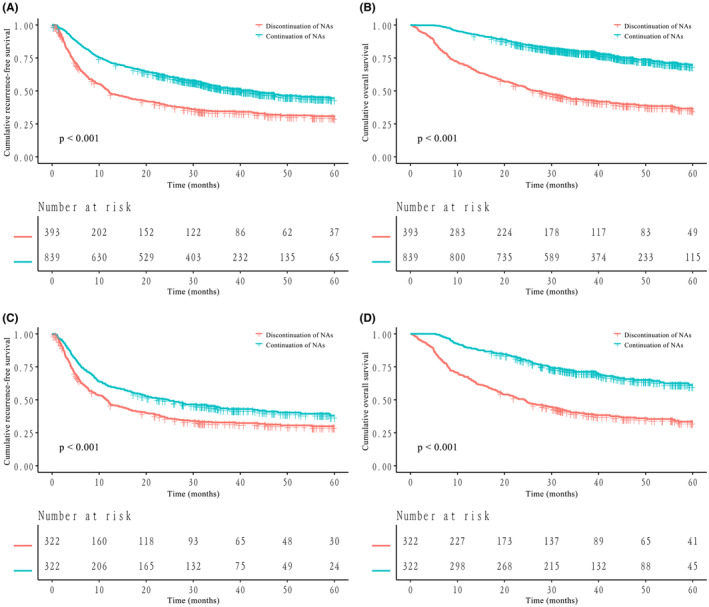
Kaplan–Meier curves for recurrence‐free survival (RFS) and overall survival (OS) of all hepatocellular carcinoma (HCC) patients stratified by nucleos(t)ide analogue (NA) therapy before and after propensity score matching (PSM). (A) RFS of all patients before PSM; (B) OS of all patients before PSM; (C) RFS of all patients after PSM; (D) OS of all patients after PSM.

Before PSM, the 1‐year, 3‐year and 5‐year RFS rates were 72.2%, 52.5% and 44.4% in the continuation of NAs group and 50.0%, 34.5% and 30.0% in the discontinuation of NAs group, respectively (*p* < 0.001, Figure 2A). The 1‐year, 3‐year and 5‐year OS rates were 93.9%, 79.8% and 69.7% in the continuation of NAs group and 69.2%, 43.5% and 35.9% in the discontinuation of NAs group, respectively (*p* < 0.001, Figure 2B). The results suggested that long‐term outcomes were significantly better in the continuation of NAs group than in the discontinuation of NAs group before PSM.

After PSM, the 1‐year, 3‐year and 5‐year RFS rates were 60.3%, 43.5% and 38.0% in the continuation of NAs group and 48.7%, 32.8% and 29.9% in the discontinuation of NAs group, respectively (*p* < 0.001, Figure 2C). The 1‐year, 3‐year and 5‐year OS rates were 89.8%, 71.2% and 61.2% in the continuation of NAs group and 67.4%, 39.9% and 33.4% in the discontinuation of NAs group, respectively (*p* < 0.001, Figure 2D). Similarly, long‐term outcomes were better for HCC patients in the NA continuation group than in the NA discontinuation group after PSM.

The detailed median times, 1‐year, 3‐year, and 5‐year rates and corresponding p values are shown in Table S5.

### 3.4 Impact of continuation of NAs or discontinuation of NAs on RFS and OS based on preoperative HBsAg status before and after PSM

**FIGURE 3:**
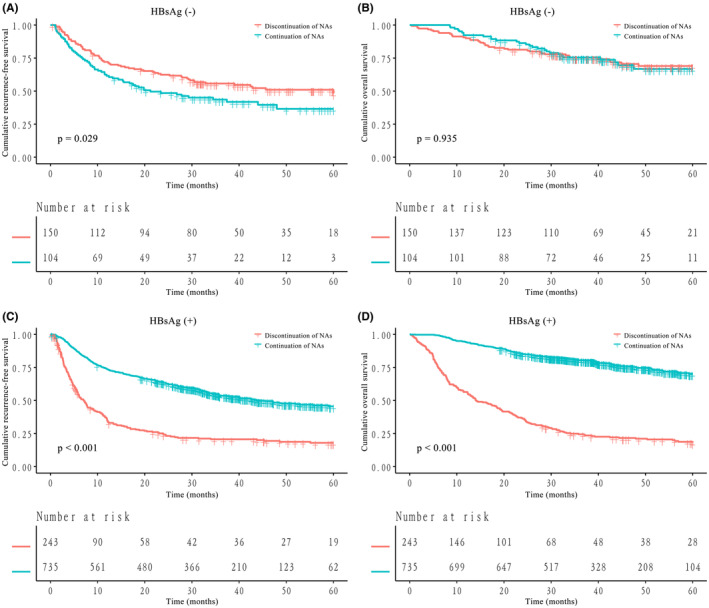
Kaplan–Meier curves for recurrence‐free survival (RFS) and overall survival (OS) of HBsAg‐negative or HBsAg‐positive hepatocellular carcinoma (HCC) patients stratified by nucleos(t)ide analogue (NA) therapy before propensity score matching (PSM). (A) RFS of HBsAg‐negative patients; (B) OS of HBsAg‐negative patients; (C) RFS of HBsAg‐positive patients; (D) OS of HBsAg‐positive patients.

Before PSM, among HBsAg‐negative patients, the RFS in the discontinuation of NAs group was significantly better than that in the continuation of NAs group (1‐year, 3‐year and 5‐year RFS, 72.1%, 55.7% and 48.2% vs. 61.5%, 43.6% and 36.6%, *p* = 0.029, Figure 3A). There were no significant differences between the OS of HBsAg‐negative patients in the discontinuation of NAs group and in the continuation of NAs group (1‐year, 3‐year and 5‐year OS, 90.0%, 75.2% and 69.0% vs. 92.3%, 75.4% and 66.8%, *p* = 0.935, Figure 3B). For HBsAg‐positive patients, the RFS and OS in the continuation of NAs group were significantly better than those in the discontinuation of NAs group (1‐year, 3‐year and 5‐year RFS, 73.7%, 53.8% and 45.5% vs. 35.8%, 20.6% and 17.9%, *p* < 0.001, Figure 3C; 1‐year, 3‐year and 5‐year OS, 94.2%, 80.4% and 70.1% vs. 56.4%, 24.4% and 18.0%, *p* < 0.001, Figure 3D). Kaplan–Meier curves for RFS and OS of all the subgroups before PSM are shown in Figure S1A,B, respectively (both *p* < 0.001).

**FIGURE 4:**
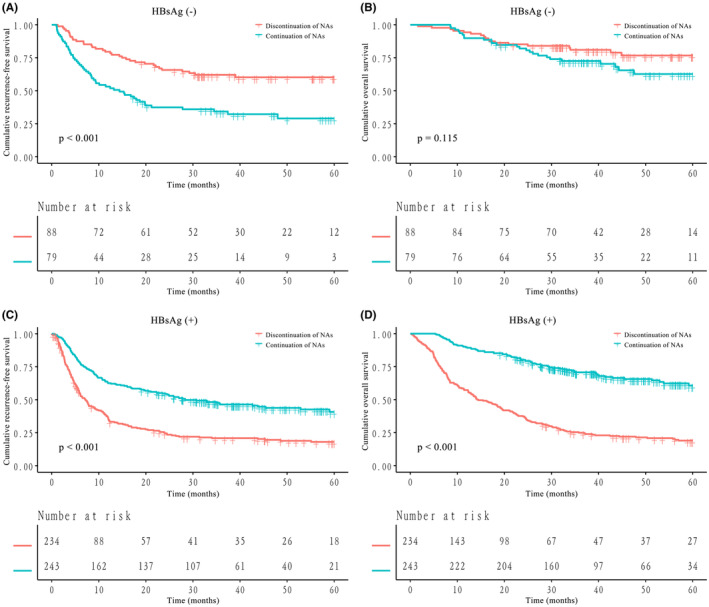
Kaplan–Meier curves for recurrence‐free survival (RFS) and overall survival (OS) of HBsAg‐negative or HBsAg‐positive hepatocellular carcinoma (HCC) patients stratified by nucleos(t)ide analogue (NA) therapy after propensity score matching (PSM). (A) RFS of HBsAg‐negative patients; (B) OS of HBsAg‐negative patients; (C) RFS of HBsAg‐positive patients; (D) OS of HBsAg‐positive patients.

After PSM, among HBsAg‐negative patients, the discontinuation of NAs group was associated with better RFS than the continuation of NAs group (1‐year, 3‐year and 5‐year RFS, 79.6%, 62.1% and 60.2% vs. 53.2%, 34.3% and 29.0%, *p* < 0.001, Figure 4A). There were also no significant differences between the OS of HBsAg‐negative patients in the discontinuation of NAs group and in the continuation of NAs group (1‐year, 3‐year and 5‐year OS, 94.3%, 81.0% and 76.7% vs. 89.9%, 72.6% and 62.7%, *p* = 0.115, Figure 4B). Similarly, the RFS and OS of HBsAg‐positive patients in the continuation of NAs group were significantly better than those in the discontinuation of NAs group (1‐year, 3‐year and 5‐year RFS, 62.6%, 46.4% and 40.8% vs. 36.1%, 20.9% and 18.0%, *p* < 0.001, Figure 4C; 1‐year, 3‐year and 5‐year OS, 89.7%, 70.7% and 60.6% vs. 57.3%, 24.9% and 18.9%, *p* < 0.001, Figure 4D). Kaplan–Meier curves for RFS and OS values in all subgroups after PSM are shown in Figure S1C,D, respectively (both *p* < 0.001).

The detailed median times, 1‐year, 3‐year, and 5‐year rates and corresponding p values of the above subgroup analyses are shown in Table S5.

Furthermore, PSM was specially performed for HBsAg‐negative patients to balance the differences between HBsAg‐negative patients who continued and discontinued NAs. As shown in Table S6, 70 matched pairs of patients were obtained by PSM, and there were no significant differences between the two cohorts. After PSM, HBsAg‐negative patients who discontinued NAs was associated with better RFS than those who continued NAs (1‐year, 3‐year and 5‐year RFS, 72.9%, 59.8% and 51.2% vs. 57.1%, 46.9% and 36.7%, *p* = 0.032, Figure S2A). There were no significant differences between the OS of HBsAg‐negative patients who discontinued and continued NAs (1‐year, 3‐year and 5‐year OS, 91.4%, 77.8% and 72.6% vs. 92.9%, 74.1% and 71.4%, *p* = 0.764, Figure S2B).

### 3.5 Stratified analyses and interaction tests of associations between continuation of NAs or discontinuation of NAs and long‐term outcomes based on preoperative HBsAg status before and after PSM

As shown in Table 4, stratified analyses and interaction tests were performed to further assess the interactive effect of HBsAg status on associations between the continuation of NAs or the discontinuation of NAs and long‐term outcomes in HBeAg‐negative HBV‐related HCC patients. Interaction tests indicated that HBsAg status and continuation of NAs or discontinuation of NAs played an interactive role in the RFS and OS of patients both before PSM and after PSM (all *p* for interaction <0.001). Of note, the RFS of HBsAg‐negative patients in the continuation of NAs group was significantly worse than that in the discontinuation of NAs group both before PSM (HR 1.468, 95% CI 1.038–2.075, *p* = 0.030) and after PSM (HR 2.377, 95% CI 1.544–3.658, *p* < 0.001). However, the results of OS analysis between the discontinuation of NAs group and the continuation of NAs group for HBsAg‐negative patients were not statistically significant both before PSM (*p* = 0.920) and after PSM (*p* = 0.126).

**TABLE 4:** Effect size of discontinuation of nucleos(t)ide analogues (NAs) or continuation of NAs on recurrence‐free survival (RFS) and overall survival (OS) based on preoperative hepatitis B surface antigen (HBsAg) status before and after propensity score matching (PSM).

Variables	Discontinuation of NAs			Continuation of NAs			*p* for interaction
	*N*	HR (95% CI)	*p*‐value	*N*	HR (95% CI)	*p*‐value	
*Before PSM*							
RFS							
HBsAg status							
HBsAg‐negative	150	Reference	–	104	1.468 (1.038–2.075)	**0.030**	**<0.001**
HBsAg‐positive	243	2.932 (2.220–3.873)	**<0.001**	735	1.027 (0.794–1.330)	0.838	
OS							
HBsAg status							
HBsAg‐negative	150	Reference	–	104	0.976 (0.603–1.578)	0.920	**<0.001**
HBsAg‐positive	243	4.960 (3.538–6.953)	**<0.001**	735	0.820 (0.583–1.153)	0.254	
*After PSM*							
RFS							
HBsAg status							
HBsAg‐negative	88	Reference	–	79	2.377 (1.544–3.658)	**<0.001**	**<0.001**
HBsAg‐positive	234	3.528 (2.440–5.103)	**<0.001**	243	1.619 (1.110–2.360)	**0.012**	
OS							
HBsAg status							
HBsAg‐negative	88	Reference	–	79	1.605 (0.876–2.942)	0.126	**<0.001**
HBsAg‐positive	234	6.752 (4.158–10.966)	**<0.001**	243	1.687 (1.011–2.814)	**0.045**	

Note: Bold text indicated that these variables were statistically significant.

Abbreviations: CI, confidence interval; HBsAg, hepatitis B surface antigen; HR, hazard ratio; NAs, nucleos(t)ide analogues; OS, overall survival; PSM, propensity score matching; RFS, recurrence‐free survival.

## 4 DISCUSSION

In recent years, several researchers have studied the potential for CHB patients to discontinue NA therapy from immunological and virological viewpoints.^19^, ^20^ However, from guidelines and clinical practice, there is no clearly established consensus on the criteria for discontinuation of NA therapy in HBV‐related HCC patients. Moreover, scholars in numerous studies have elucidated associations between hepatitis B seromarkers and the risk of HBV‐related HCC, but relatively few studies have focused on the role of preoperative hepatitis B seromarkers in the postoperative outcomes of HBV‐related HCC.^6^, ^21^, ^22^ To our knowledge, this is the first large‐scale historical cohort study to investigate the potential for HBeAg‐negative HBV‐related HCC patients to discontinue NA therapy from a postoperative prognostic standpoint. In the present study, we found that compared with the discontinuation of NAs, the continuation of NAs was an independent protective factor and was significantly related to a better prognosis for all enrolled patients, which was not unexpected. Intriguingly, when stratified analyses were performed based on preoperative serum HBsAg status, the continuation of NAs was demonstrated to be an independent risk factor for RFS and was associated with worse RFS in HBsAg‐negative patients. Interaction tests also confirmed a significant effect of the interaction between the continuation of NAs or the discontinuation of NAs and HBsAg status on the long‐term outcomes of HBV‐related HCC. The results were confirmed by PSM analyses and presented the potential for clinicians to discontinue NAs in HBeAg‐negative HBV‐related HCC patients who had achieved HBsAg seroclearance before surgery.

As common antiviral drugs, NAs, represented by ETV and TDF, were demonstrated to be able to dramatically inhibit viral replication and effectively decrease the incidence of HBV‐related HCC,^23^, ^24^, ^25^ which might be a major concern among CHB patients. However, HBV‐related HCC patients are more concerned about the effect of NA therapy on long‐term outcomes after HCC surgery. Fortunately, NAs were also reported to be associated with reduced HBV‐related HCC recurrence and better postoperative survival for HBsAg‐positive patients.^26^, ^27^, ^28^ Nevertheless, NA therapy is not a perfect antiviral treatment for HBV patients thus far. Some younger patients or those planning pregnancy may be reluctant to receive life‐long antiviral treatment when considering safety issues and possible adverse effects.^29^ Moreover, infinite treatment may lead to reduced patient compliance and may not be conducive to improved cost‐effectiveness. Therefore, in recent years, an increasing number of scholars have begun to focus on the indication and feasibility of NA discontinuation for CHB patients.^30^, ^31^, ^32^ A multicenter study from Korea indicated that for CHB patients who achieved HBsAg seroclearance with NAs, the discontinuation of NAs was not associated with an increased risk of HCC development.^30^ Another longitudinal cohort study including Caucasians and Asians suggested that discontinuation of long‐term NA therapy was not associated with higher HCC risk for noncirrhotic HBeAg‐negative CHB patients.^31^ However, associations between the continuation of NAs or the discontinuation of NAs and postoperative prognosis have hardly been reported to provide guidance on NA discontinuation for HBV‐related HCC patients, which prompted us to conduct the present study.

We only included preoperative HBeAg‐negative HBV‐related HCC patients during the study. This is because the phases of chronic HBV infection are generally divided based on HBeAg status.^3^, ^9^ Furthermore, HBeAg seroclearance has often been regarded as an important milestone during antiviral treatment and is associated with good prognosis in CHB patients.^12^, ^22^, ^33^ It would be of great practical and theoretical importance to focus on HBeAg‐negative patients. In our entire cohort, the continuation of NAs group accounted for the majority of patients (68.1%), reflecting a relatively conservative antiviral treatment strategy for HBV‐related HCC patients. This proportion was similar to that observed in a previous study from Asia, although the inclusion and exclusion criteria of the two studies were not fully consistent.^14^ We found that patients in the continuation of NAs group had more incidents of positive HBsAg status, fewer incidents of positive HBsAb status and a higher percentage of HBV DNA > 10^3^ IU/mL in preoperative assessment, which might explain why they continued NA therapy. Considering the imbalanced baseline characteristics between the two crude groups, PSM was used to create statistically matched pairs in the continuation of NAs group and the discontinuation of NAs group. A strict caliper width equal to 0.01 of the SD for PSM guarantees good comparability between the two groups after PSM.

When we performed multivariate Cox regression analyses and log‐rank tests for comparisons of Kaplan–Meier curves in all HCC patients before and after PSM, we found that the continuation of NAs was always an independent protective factor for RFS and OS and that patients continuing NA therapy had better long‐term outcomes. These results are expected and similar to the conclusions from three previous randomized controlled trials (RCTs).^26^, ^27^, ^28^ However, the three RCTs only included HBsAg‐positive HBV‐related HCC patients and could not sufficiently conduct subgroup analyses due to limited sample size. Given that loss of HBsAg is the optimal treatment endpoint of CHB patients and that positive HBsAg status was an independent risk factor for OS both before and after PSM, subgroup analyses were subsequently conducted based on preoperative HBsAg status in more detail. Notably, the continuation of NAs was associated with a better long‐term prognosis in HBsAg‐positive HBV‐related HCC patients, whereas it seemed to play an almost opposite role in HBsAg‐negative patients. No matter before or after PSM, the continuation of NAs was an independent risk factor for RFS and was also not a protective factor for OS in HBsAg‐negative patients. Furthermore, HBsAg‐negative patients continuing NA therapy achieved worse RFS and comparable OS when compared with those discontinuing NA therapy both before and after PSM, regardless of PSM for all patients or PSM for HBsAg‐negative patients. These findings may provide a potential for preoperative HBsAg‐negative HBV‐related HCC patients to discontinue NA therapy under strict monitoring and may suggest a novel individualized therapeutic strategy to further improve the postoperative prognosis of HBV‐related HCC patients.

However, the reasons for the results in this study are not fully understood, and we can only speculate as to the mechanisms underlying the clinical findings. On the one hand, these findings may be attributed to successful viral control contributed by HBV‐specific immune T‐cell responses after antiviral treatment discontinuation.^20^, ^34^, ^35^ On the other hand, continuation of NAs for HBsAg‐negative patients may not always be beneficial due to potential side effects of long‐term medication.^29^, ^36^, ^37^ Therefore, our results should be interpreted with caution, and clinicians should fully weigh the benefits and risks of NA discontinuation in patients with HBV.

Another feature of the present study is the significant interaction between preoperative HBsAg status and continuation of NAs or discontinuation of NAs. As an effect modifier, HBsAg status markedly modified the effects of the continuation or discontinuation of NAs on the long‐term outcomes of HBV‐related HCC patients, which is a finding that has rarely been reported before. The results are understandable given that HBV replication can be suppressed by antiviral treatment and continuation or discontinuation of NAs is largely dependent on HBsAg status.^3^, ^38^ Clinicians should notice that the combined effects of HBsAg status and continuation or discontinuation of NAs may be greater than additive and realize the importance of individualized treatment.

The present study has several limitations. First, there were some potential confounding factors and unavoidable biases due to the retrospective nature of the study. To resolve this issue, we applied strict inclusion and exclusion criteria and used PSM, multivariable models and sensitivity analyses to minimize the confounding effects. Nevertheless, more prospective and randomized studies are warranted to confirm our findings and further develop well‐recognized discontinuation criteria for NA therapy. Second, this was a single‐center study only including HBV‐related HCC patients from China. Although we tried to keep the sample size as large as possible, the conclusions may not reflect the clinical practice of other medical centers or other ethnic groups. Third, considering that patients with virological relapse or HBV reactivation before surgery were a highly complex population and might require more complicated treatment strategies, we excluded them in the study. Effective strategies for this population would be an important topic for future studies. Fourth, despite our efforts, there were limited postoperative follow‐up data on HBV serological and virological indicators. Thus, we were unable to further analyze virological relapse after surgery. Detailed information on cause of death was also not available for all patients and analyses to assess the competing risk of death were not performed. Fifth, the median survival in some subgroups was not reached because of their relatively good prognosis and relatively short follow‐up times. We will conduct more studies targeting the correlated patients with longer follow‐up times in the future. Finally, the current study focused specifically on the interaction between HBsAg status and the continuation or discontinuation of NAs. Further studies are needed to better understand the interactions between the continuation or discontinuation of NAs and other key factors of HBV‐related HCC.

Taken together, among NA‐treated HBeAg‐negative patients who underwent curative liver resection for HBV‐related HCC, NA discontinuation was associated with better RFS and comparable OS than NA continuation in preoperative HBsAg‐negative patients. For HBsAg‐positive patients, NA continuation remained associated with better survival. From the perspective of postoperative prognosis, these results represent an important step in exploring the feasibility of NA discontinuation for those who have achieved HBsAg seroclearance before surgery and suggest the potential of discontinuing antiviral treatment with NAs in selected patients under conditions of strict monitoring.

## AUTHOR CONTRIBUTIONS

**Ting Sun:** Conceptualization (lead); data curation (lead); formal analysis (lead); writing – original draft (lead). **Yiwen Qiu:** Conceptualization (supporting); data curation (supporting); formal analysis (supporting); writing – original draft (supporting). **Tao Wang:** Data curation (supporting); investigation (equal); methodology (equal). **Yi Yang:** Data curation (supporting); investigation (equal); methodology (equal). **Haizhou Qiu:** Visualization (equal). **Shu Shen:** Visualization (equal). **Huasheng Pang:** Visualization (equal). **Wentao Wang:** Funding acquisition (equal); project administration (equal); supervision (equal).

## FUNDING INFORMATION

This research was supported by the Science and Technology Program of Sichuan Science and Technology Department (No. 2019YFS0576 & No. 2023YFS0229), the National Natural Science Foundation of China (No. 82170543 & No. 82000599), Health Commission of the Tibet Autonomous Region (No. 311220432) and NHC Key Laboratory of Echinococcosis Prevention and Control (No. 2021WZK1004). Corresponding author Wentao Wang is the guarantor. The funding body financed the costs of the study and contributed to the design of the study, interpretation of data, and revising the manuscript.

## CONFLICT OF INTEREST STATEMENT

The authors report no conflict of interest.

## ETHICS STATEMENT

The study was conducted in accordance with the Declaration of Helsinki and was approved by the Ethics Committee of West China Hospital of Sichuan University (No. 2021–1151). The requirement for informed consent was waived because the study was retrospective and the data were anonymized.

## Supporting information


Figure S1.

Figure S2.

Table S1.

Table S2.

Table S3.

Table S4.

Table S5.

Table S6.

## Data Availability

The data analyzed during the current study are available from the corresponding author upon reasonable request.
