# Supplementary figures and images for: Effect of nucleos(t)ide analogue discontinuation on the prognosis of HBeAg‐negative hepatitis B virus‐related hepatocellular carcinoma after hepatectomy: A propensity score matching analysis
Source: Cancer Med. 2024 Sep 1;13(16):e70185. doi: 10.1002/cam4.70185 (PMC11366777; doi:10.1002/cam4.70185)

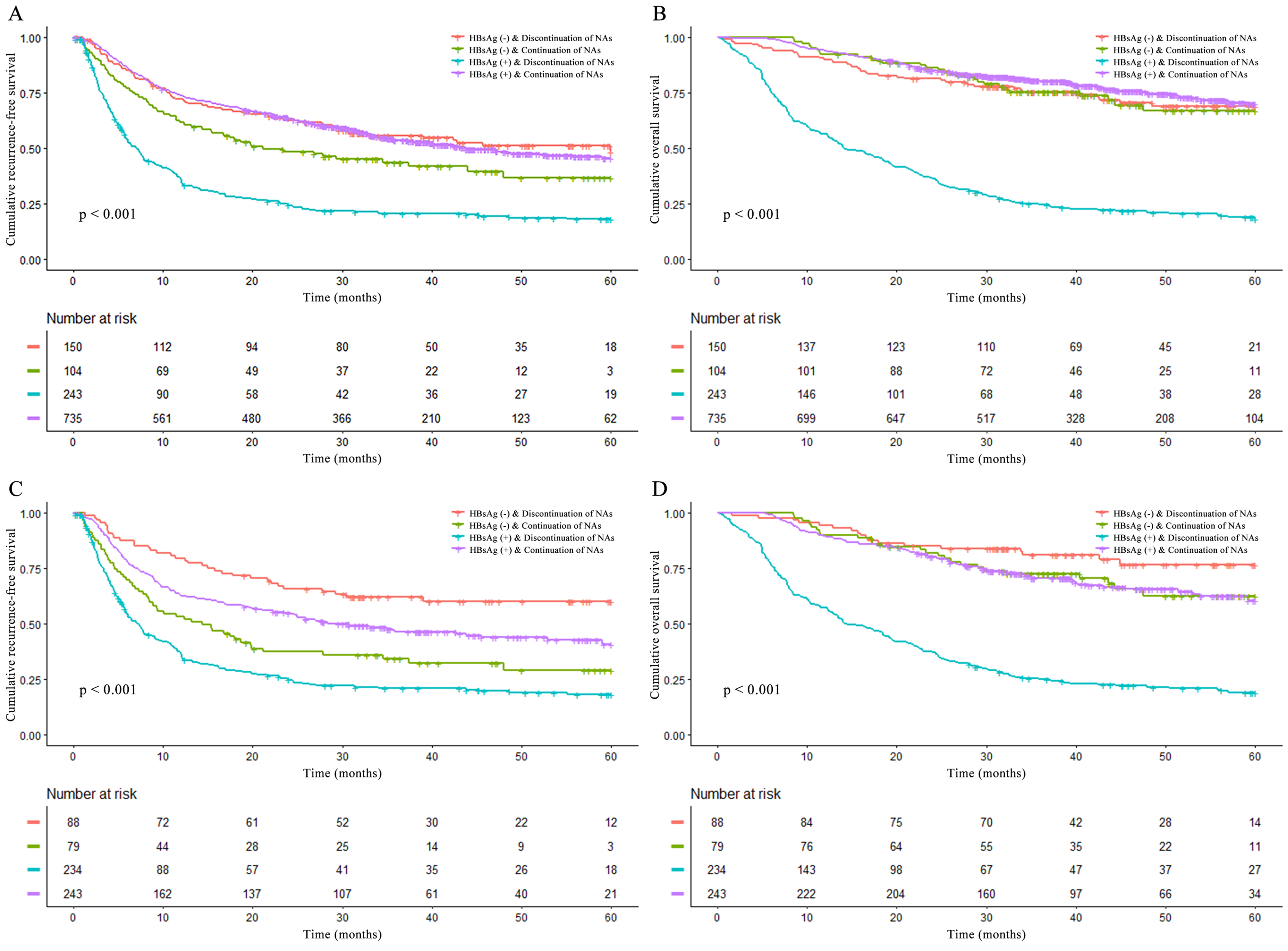

Supplement: Supplementary file 1 — Figure S1. [file CAM4-13-e70185-s005.tif]

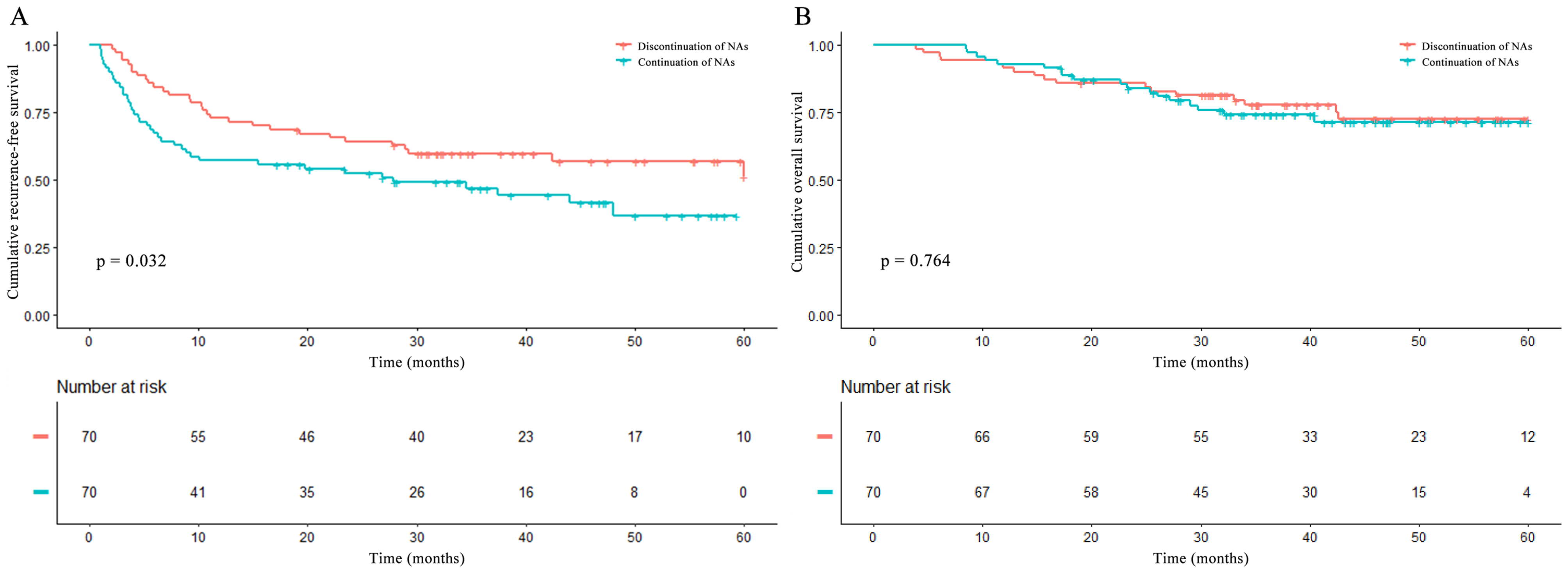

Supplement: Supplementary file 2 — Figure S2. [file CAM4-13-e70185-s002.tif]
