# Supplementary material for: Effect of nucleos(t)ide analogue discontinuation on the prognosis of HBeAg‐negative hepatitis B virus‐related hepatocellular carcinoma after hepatectomy: A propensity score matching analysis
Source: Cancer Med. 2024 Sep 1;13(16):e70185. doi: 10.1002/cam4.70185 (PMC11366777; doi:10.1002/cam4.70185)
Supplement: Supplementary file 3 — Table S1. [file CAM4-13-e70185-s006.docx]

**Table S1.** **Univariate and multivariate Cox regression analysis of recurrence-free survival (RFS) and overall survival (OS) in hepatitis B surface antigen (HBsAg)-negative hepatocellular carcinoma (HCC) patients before propensity score matching (PSM)**

| **Variables** | **RFS** | | | | **OS** | | | |
| --- | --- | --- | --- | --- | --- | --- | --- | --- |
|  | **Univariate** | | **Multivariate** | | **Univariate** | | **Multivariate** | |
|  | **HR (95% CI)** | **P value** | **HR (95% CI)** | **P value** | **HR (95% CI)** | **P value** | **HR (95% CI)** | **P value** |
| Age, years | 1.007 (0.990-1.024) | 0.432 |  |  | 0.990 (0.968-1.013) | 0.388 |  |  |
| Male sex | 1.927 (0.898-4.134) | 0.092 |  |  | 1.645 (0.599-4.523) | 0.334 |  |  |
| BMI, kg/m^2^ | 0.958 (0.908-1.010) | 0.111 |  |  | 0.878 (0.813-0.948) | **< 0.001** | 1.067 (0.969-1.176) | 0.188 |
| Alcohol consumption | 1.303 (0.922-1.841) | 0.133 |  |  | 1.914 (1.180-3.105) | **0.009** | 0.987 (0.531-1.832) | 0.966 |
| Cigarette smoking | 1.904 (1.310-2.768) | **< 0.001** | 1.784 (1.150-2.767) | **0.010** | 2.654 (1.498-4.704) | **< 0.001** | 2.042 (0.948-4.397) | 0.068 |
| Diabetes mellitus | 1.213 (0.717-2.052) | 0.472 |  |  | 1.373 (0.701-2.688) | 0.355 |  |  |
| Hypertension | 1.023 (0.701-1.495) | 0.905 |  |  | 0.860 (0.507-1.459) | 0.577 |  |  |
| ETV monotherapy | 1.292 (0.823-2.028) | 0.266 |  |  | 0.995 (0.553-1.789) | 0.986 |  |  |
| NAs, continuation vs. discontinuation | 1.468 (1.037-2.077) | **0.030** | 2.047 (1.348-3.109) | **< 0.001** | 0.980 (0.606-1.585) | 0.935 |  |  |
| HBsAb-positive | 0.747 (0.522-1.067) | 0.109 |  |  | 0.984 (0.593-1.633) | 0.950 |  |  |
| HBeAb-positive | 1.008 (0.700-1.451) | 0.968 |  |  | 1.002 (0.607-1.654) | 0.993 |  |  |
| HBV DNA, IU/mL, > 10^3^ vs. ≤ 10^3^ | 1.178 (0.723-1.917) | 0.511 |  |  | 1.594 (0.871-2.920) | 0.131 |  |  |
| AFP, ng/mL, > 400 vs. ≤ 400 | 1.541 (1.081-2.197) | **0.017** | 1.553 (0.985-2.451) | 0.058 | 3.197 (1.984-5.152) | **< 0.001** | 2.048 (1.119-3.750) | **0.020** |
| Hemoglobin, g/L | 0.982 (0.973-0.991) | **< 0.001** | 0.988 (0.977-0.999) | **0.030** | 0.981 (0.970-0.993) | **0.001** | 0.991 (0.978-1.004) | 0.184 |
| Platelets, 10^9^/L | 1.006 (1.004-1.009) | **< 0.001** | 1.005 (1.002-1.007) | **< 0.001** | 1.008 (1.005-1.011) | **< 0.001** | 1.003 (0.999-1.007) | 0.113 |
| ALT, IU/L | 1.004 (1.001-1.007) | **0.021** | 1.008 (0.999-1.017) | 0.093 | 1.005 (1.000-1.009) | **0.042** | 1.007 (0.996-1.019) | 0.192 |
| AST, IU/L | 1.008 (1.004-1.012) | **< 0.001** | 0.996 (0.983-1.009) | 0.570 | 1.010 (1.005-1.015) | **< 0.001** | 0.993 (0.976-1.009) | 0.373 |
| TBIL, μmol/L | 0.961 (0.931-0.993) | **0.016** | 1.000 (0.965-1.036) | 0.995 | 0.957 (0.914-1.001) | 0.054 |  |  |
| Albumin, g/L | 0.908 (0.868-0.949) | **< 0.001** | 0.997 (0.947-1.049) | 0.902 | 0.840 (0.790-0.894) | **< 0.001** | 0.904 (0.837-0.976) | **0.010** |
| PT, s | 0.974 (0.877-1.082) | 0.626 |  |  | 1.037 (0.915-1.176) | 0.568 |  |  |
| Child‒Pugh grade, A vs. B | 1.554 (0.384-6.288) | 0.537 |  |  | 1.391 (0.193-10.034) | 0.743 |  |  |
| ASA grade, Ⅱ vs. Ⅰ | 0.959 (0.678-1.358) | 0.815 |  |  | 0.948 (0.590-1.523) | 0.824 |  |  |
| Blood loss, mL | 1.000 (1.000-1.000) | **0.012** | 1.000 (1.000-1.000) | 0.942 | 1.000 (1.000-1.000) | **< 0.001** | 1.000 (1.000-1.001) | **0.045** |
| Operation time, min | 1.002 (1.000-1.004) | 0.071 |  |  | 1.004 (1.001-1.006) | **0.006** | 1.000 (0.996-1.004) | 0.921 |
| Blood transfusion | 2.029 (1.163-3.538) | **0.013** | 1.277 (0.549-2.971) | 0.570 | 2.347 (1.164-4.733) | **0.017** | 0.684 (0.180-2.595) | 0.577 |
| Anatomic resection | 0.647 (0.438-0.955) | **0.028** | 0.502 (0.320-0.787) | **0.003** | 0.915 (0.551-1.519) | 0.731 |  |  |
| Single tumor | 0.671 (0.385-1.169) | 0.159 |  |  | 0.581 (0.297-1.137) | 0.113 |  |  |
| Tumor size, cm, > 5 vs. ≤ 5 | 2.121 (1.483-3.034) | **< 0.001** | 2.102 (1.350-3.272) | **0.001** | 2.385 (1.437-3.959) | **< 0.001** | 1.876 (0.995-3.537) | 0.052 |
| MVI | 1.699 (1.187-2.430) | **0.004** | 1.522 (1.002-2.313) | **0.049** | 2.502 (1.557-4.023) | **< 0.001** | 2.160 (1.169-3.992) | **0.014** |
| Satellite nodule | 3.853 (2.500-5.937) | **< 0.001** | 3.502 (2.152-5.699) | **< 0.001** | 1.857 (1.032-3.342) | **0.039** | 1.331 (0.640-2.766) | 0.444 |
| PVTT | 1.546 (0.784-3.051) | 0.209 |  |  | 3.293 (1.632-6.647) | **< 0.001** | 1.909 (0.690-5.281) | 0.213 |
| Cirrhosis | 0.714 (0.484-1.052) | 0.089 |  |  | 0.493 (0.274-0.886) | **0.018** | 0.696 (0.346-1.400) | 0.309 |
| Edmondson-Steiner grade, ≥ Ⅲ vs. ≤ Ⅱ | 1.610 (1.138-2.279) | **0.007** | 1.058 (0.700-1.600) | 0.789 | 2.169 (1.349-3.486) | **0.001** | 1.492 (0.827-2.691) | 0.184 |
| BCLC stage, 0/A vs. B/C | 0.402 (0.261-0.620) | **< 0.001** | 0.573 (0.351-0.935) | **0.026** | 0.275 (0.165-0.456) | **< 0.001** | 0.542 (0.282-1.043) | 0.067 |

Bold text indicated that these variables were statistically significant.

Abbreviations: RFS, recurrence-free survival; OS, overall survival; HBsAg, hepatitis B surface antigen; HCC, hepatocellular carcinoma; PSM, propensity score matching; HR, hazard ratio; CI, confidence interval; BMI, body mass index; ETV, entecavir; NAs, nucleos(t)ide analogues; HBsAb, hepatitis B surface antibody; HBeAb, hepatitis B e antibody; HBV, hepatitis B virus; AFP, alpha-fetoprotein; ALT, alanine aminotransferase; AST, aspartate aminotransferase; TBIL, total bilirubin; PT, prothrombin time; ASA, American Society of Anesthesiologists; MVI, microvascular invasion; PVTT, portal vein tumor thrombus; BCLC, Barcelona Clinic Liver Cancer
