# Supplementary material for: Effect of nucleos(t)ide analogue discontinuation on the prognosis of HBeAg‐negative hepatitis B virus‐related hepatocellular carcinoma after hepatectomy: A propensity score matching analysis
Source: Cancer Med. 2024 Sep 1;13(16):e70185. doi: 10.1002/cam4.70185 (PMC11366777; doi:10.1002/cam4.70185)
Supplement: Supplementary file 4 — Table S2. [file CAM4-13-e70185-s007.docx]

**Table S2. Univariate and multivariate Cox regression analysis of recurrence-free survival (RFS) and overall survival (OS) in hepatitis B surface antigen (HBsAg)-positive hepatocellular carcinoma (HCC) patients before propensity score matching (PSM)**

| **Variables** | **RFS** | | | | **OS** | | | |
| --- | --- | --- | --- | --- | --- | --- | --- | --- |
|  | **Univariate** | | **Multivariate** | | **Univariate** | | **Multivariate** | |
|  | **HR (95% CI)** | **P value** | **HR (95% CI)** | **P value** | **HR (95% CI)** | **P value** | **HR (95% CI)** | **P value** |
| Age, years | 0.988 (0.980-0.996) | **0.002** | 0.987 (0.979-0.995) | **0.002** | 0.983 (0.974-0.992) | **< 0.001** | 0.987 (0.976-0.998) | **0.025** |
| Male sex | 1.083 (0.851-1.379) | 0.517 |  |  | 1.155 (0.857-1.557) | 0.343 |  |  |
| BMI, kg/m^2^ | 1.003 (0.977-1.030) | 0.837 |  |  | 0.996 (0.965-1.029) | 0.824 |  |  |
| Alcohol consumption | 0.956 (0.802-1.138) | 0.611 |  |  | 1.017 (0.824-1.256) | 0.873 |  |  |
| Cigarette smoking | 1.030 (0.869-1.221) | 0.732 |  |  | 1.180 (0.961-1.450) | 0.115 |  |  |
| Diabetes mellitus | 0.975 (0.692-1.374) | 0.887 |  |  | 1.145 (0.778-1.683) | 0.492 |  |  |
| Hypertension | 0.813 (0.633-1.046) | 0.108 |  |  | 0.691 (0.496-0.964) | **0.029** | 1.155 (0.755-1.766) | 0.507 |
| ETV monotherapy | 1.082 (0.872-1.341) | 0.475 |  |  | 1.179 (0.900-1.544) | 0.232 |  |  |
| NAs, continuation vs. discontinuation | 0.350 (0.292-0.419) | **< 0.001** | 0.419 (0.347-0.507) | **< 0.001** | 0.166 (0.135-0.205) | **< 0.001** | 0.190 (0.153-0.237) | **< 0.001** |
| HBsAb-positive | 1.291 (0.928-1.797) | 0.129 |  |  | 1.134 (0.750-1.717) | 0.551 |  |  |
| HBeAb-positive | 1.275 (0.887-1.833) | 0.189 |  |  | 2.217 (1.247-3.941) | **0.007** | 1.187 (0.656-2.147) | 0.572 |
| HBV DNA, IU/mL, > 10^3^ vs. ≤ 10^3^ | 1.309 (1.104-1.552) | **0.002** | 1.064 (0.890-1.273) | 0.497 | 1.520 (1.235-1.872) | **< 0.001** | 1.166 (0.933-1.457) | 0.177 |
| AFP, ng/mL, > 400 vs. ≤ 400 | 1.915 (1.615-2.271) | **< 0.001** | 1.393 (1.158-1.676) | **< 0.001** | 2.316 (1.884-2.847) | **< 0.001** | 1.543 (1.233-1.930) | **< 0.001** |
| Hemoglobin, g/L | 0.995 (0.990-1.000) | **0.035** | 0.997 (0.992-1.002) | 0.233 | 0.998 (0.992-1.004) | 0.522 |  |  |
| Platelets, 10^9^/L | 1.002 (1.001-1.004) | **< 0.001** | 0.999 (0.998-1.000) | 0.065 | 1.003 (1.002-1.004) | **< 0.001** | 0.998 (0.997-1.000) | **0.037** |
| ALT, IU/L | 1.001 (1.000-1.002) | 0.096 |  |  | 1.001 (1.000-1.003) | **0.030** | 0.998 (0.995-1.001) | 0.186 |
| AST, IU/L | 1.002 (1.001-1.003) | **< 0.001** | 1.000 (0.998-1.001) | 0.608 | 1.002 (1.001-1.004) | **< 0.001** | 1.003 (0.999-1.006) | 0.175 |
| TBIL, μmol/L | 1.006 (0.996-1.015) | 0.230 |  |  | 1.012 (1.006-1.018) | **< 0.001** | 1.008 (1.003-1.014) | **0.003** |
| Albumin, g/L | 0.948 (0.930-0.968) | **< 0.001** | 0.971 (0.948-0.993) | **0.012** | 0.941 (0.918-0.964) | **< 0.001** | 0.974 (0.948-1.001) | 0.058 |
| PT, s | 1.111 (1.029-1.198) | **0.007** | 1.010 (0.933-1.094) | 0.801 | 1.165 (1.065-1.275) | **< 0.001** | 1.041 (0.945-1.147) | 0.413 |
| Child‒Pugh grade, A vs. B | 1.326 (0.426-4.127) | 0.626 |  |  | 0.872 (0.280-2.718) | 0.813 |  |  |
| ASA grade, Ⅱ vs. Ⅰ | 0.950 (0.790-1.142) | 0.585 |  |  | 0.784 (0.622-0.988) | **0.039** | 0.911 (0.662-1.254) | 0.568 |
| Blood loss, mL | 1.000 (1.000-1.000) | **< 0.001** | 1.000 (1.000-1.000) | 0.595 | 1.000 (1.000-1.000) | **< 0.001** | 1.000 (0.999-1.000) | 0.193 |
| Operation time, min | 1.003 (1.002-1.004) | **< 0.001** | 1.001 (1.000-1.002) | 0.108 | 1.004 (1.003-1.005) | **< 0.001** | 1.001 (0.999-1.002) | 0.238 |
| Blood transfusion | 1.510 (1.085-2.101) | **0.014** | 0.953 (0.633-1.435) | 0.817 | 1.800 (1.252-2.589) | **0.002** | 1.294 (0.804-2.083) | 0.289 |
| Anatomic resection | 1.215 (1.020-1.449) | **0.029** | 0.910 (0.756-1.097) | 0.324 | 1.228 (0.994-1.516) | 0.057 |  |  |
| Single tumor | 0.536 (0.435-0.660) | **< 0.001** | 0.638 (0.440-0.926) | **0.018** | 0.576 (0.451-0.737) | **< 0.001** | 0.843 (0.532-1.335) | 0.466 |
| Tumor size, cm, > 5 vs. ≤ 5 | 2.966 (2.489-3.536) | **< 0.001** | 2.120 (1.726-2.605) | **< 0.001** | 3.155 (2.535-3.927) | **< 0.001** | 1.766 (1.373-2.272) | **< 0.001** |
| MVI | 2.237 (1.875-2.670) | **< 0.001** | 1.428 (1.175-1.736) | **< 0.001** | 2.597 (2.107-3.201) | **< 0.001** | 1.482 (1.169-1.879) | **0.001** |
| Satellite nodule | 2.356 (1.839-3.017) | **< 0.001** | 1.511 (1.160-1.969) | **0.002** | 2.266 (1.710-3.003) | **< 0.001** | 1.351 (0.991-1.841) | 0.057 |
| PVTT | 2.962 (2.281-3.846) | **< 0.001** | 1.314 (0.837-2.060) | 0.235 | 3.877 (2.954-5.087) | **< 0.001** | 1.188 (0.698-2.023) | 0.526 |
| Cirrhosis | 1.085 (0.915-1.288) | 0.348 |  |  | 1.074 (0.873-1.321) | 0.500 |  |  |
| Edmondson-Steiner grade, ≥ Ⅲ vs. ≤ Ⅱ | 1.529 (1.289-1.813) | **< 0.001** | 1.280 (1.069-1.531) | **0.007** | 1.884 (1.528-2.322) | **< 0.001** | 1.561 (1.249-1.951) | **< 0.001** |
| BCLC stage, 0/A vs. B/C | 0.371 (0.306-0.450) | **< 0.001** | 0.940 (0.609-1.452) | 0.781 | 0.318 (0.256-0.395) | **< 0.001** | 0.656 (0.385-1.119) | 0.122 |

Bold text indicated that these variables were statistically significant.

Abbreviations: RFS, recurrence-free survival; OS, overall survival; HBsAg, hepatitis B surface antigen; HCC, hepatocellular carcinoma; PSM, propensity score matching; HR, hazard ratio; CI, confidence interval; BMI, body mass index; ETV, entecavir; NAs, nucleos(t)ide analogues; HBsAb, hepatitis B surface antibody; HBeAb, hepatitis B e antibody; HBV, hepatitis B virus; AFP, alpha-fetoprotein; ALT, alanine aminotransferase; AST, aspartate aminotransferase; TBIL, total bilirubin; PT, prothrombin time; ASA, American Society of Anesthesiologists; MVI, microvascular invasion; PVTT, portal vein tumor thrombus; BCLC, Barcelona Clinic Liver Cancer
