# Supplementary material for: Effect of nucleos(t)ide analogue discontinuation on the prognosis of HBeAg‐negative hepatitis B virus‐related hepatocellular carcinoma after hepatectomy: A propensity score matching analysis
Source: Cancer Med. 2024 Sep 1;13(16):e70185. doi: 10.1002/cam4.70185 (PMC11366777; doi:10.1002/cam4.70185)
Supplement: Supplementary file 5 — Table S3. [file CAM4-13-e70185-s004.docx]

**Table S3.** **Univariate and multivariate Cox regression analysis of recurrence-free survival (RFS) and overall survival (OS) in hepatitis B surface antigen (HBsAg)-negative hepatocellular carcinoma (HCC) patients after propensity score matching (PSM)**

| **Variables** | **RFS** | | | | **OS** | | | |
| --- | --- | --- | --- | --- | --- | --- | --- | --- |
|  | **Univariate** | | **Multivariate** | | **Univariate** | | **Multivariate** | |
|  | **HR (95% CI)** | **P value** | **HR (95% CI)** | **P value** | **HR (95% CI)** | **P value** | **HR** **(95% CI)** | **P value** |
| Age, years | 1.006 (0.984-1.029) | 0.599 |  |  | 0.977 (0.948-1.007) | 0.135 |  |  |
| Male sex | 2.219 (0.899-5.480) | 0.084 |  |  | 1.603 (0.495-5.188) | 0.431 |  |  |
| BMI, kg/m^2^ | 0.921 (0.860-0.986) | **0.018** | 1.036 (0.938-1.144) | 0.491 | 0.831 (0.749-0.922) | **< 0.001** | 0.869 (0.739-1.023) | 0.092 |
| Alcohol consumption | 1.370 (0.899-2.088) | 0.143 |  |  | 2.588 (1.366-4.901) | **0.004** | 0.603 (0.218-1.664) | 0.328 |
| Cigarette smoking | 1.875 (1.190-2.956) | **0.007** | 2.745 (1.392-5.413) | **0.004** | 2.847 (1.365-5.936) | **0.005** | 6.556 (2.101-20.457) | **0.001** |
| Diabetes mellitus | 0.991 (0.558-1.759) | 0.974 |  |  | 1.478 (0.707-3.089) | 0.299 |  |  |
| Hypertension | 1.001 (0.643-1.559) | 0.997 |  |  | 0.750 (0.391-1.438) | 0.386 |  |  |
| ETV monotherapy | 1.269 (0.747-2.157) | 0.378 |  |  | 0.928 (0.457-1.884) | 0.837 |  |  |
| NAs, continuation vs. discontinuation | 2.407 (1.561-3.711) | **< 0.001** | 2.756 (1.537-4.942) | **< 0.001** | 1.620 (0.883-2.969) | 0.119 |  |  |
| HBsAb-positive | 0.645 (0.417-0.998) | **0.049** | 0.543 (0.316-0.933) | **0.027** | 0.547 (0.298-1.002) | 0.051 |  |  |
| HBeAb-positive | 0.828 (0.540-1.270) | 0.388 |  |  | 0.848 (0.460-1.563) | 0.596 |  |  |
| HBV DNA, IU/mL, > 10^3^ vs. ≤ 10^3^ | 0.898 (0.507-1.592) | 0.714 |  |  | 1.318 (0.630-2.756) | 0.464 |  |  |
| AFP, ng/mL, > 400 vs. ≤ 400 | 2.006 (1.292-3.115) | **0.002** | 1.822 (1.081-3.073) | **0.024** | 4.296 (2.350-7.853) | **< 0.001** | 14.278 (5.101-39.961) | **< 0.001** |
| Hemoglobin, g/L | 0.980 (0.969-0.990) | **< 0.001** | 0.991 (0.976-1.006) | 0.245 | 0.978 (0.964-0.992) | **0.002** | 1.044 (1.013-1.076) | **0.006** |
| Platelets, 10^9^/L | 1.005 (1.003-1.008) | **< 0.001** | 1.001 (0.998-1.005) | 0.446 | 1.009 (1.006-1.013) | **< 0.001** | 1.000 (0.993-1.008) | 0.925 |
| ALT, IU/L | 1.010 (1.000-1.019) | **0.039** | 1.002 (0.986-1.019) | 0.770 | 1.011 (0.997-1.025) | 0.120 |  |  |
| AST, IU/L | 1.021 (1.012-1.030) | **< 0.001** | 1.029 (1.010-1.048) | **0.003** | 1.032 (1.020-1.044) | **< 0.001** | 1.050 (1.027-1.072) | **< 0.001** |
| TBIL, μmol/L | 0.970 (0.934-1.007) | 0.113 |  |  | 0.982 (0.930-1.036) | 0.500 |  |  |
| Albumin, g/L | 0.900 (0.851-0.951) | **< 0.001** | 1.007 (0.926-1.094) | 0.877 | 0.807 (0.742-0.877) | **< 0.001** | 0.906 (0.785-1.046) | 0.179 |
| PT, s | 1.038 (0.845-1.274) | 0.725 |  |  | 1.245 (0.962-1.610) | 0.095 |  |  |
| Child‒Pugh grade, A vs. B | 0.115 (0.015-0.860) | **0.035** | 0.060 (0.004-0.882) | **0.040** | 0.006 (0.000-0.097) | < 0.001 | -^*^ | -^*^ |
| ASA grade, Ⅱ vs. Ⅰ | 0.968 (0.628-1.494) | 0.885 |  |  | 0.748 (0.410-1.366) | 0.345 |  |  |
| Blood loss, mL | 1.000 (1.000-1.001) | **0.029** | 1.000 (0.999-1.000) | 0.214 | 1.000 (1.000-1.000) | **0.003** | 1.000 (0.999-1.000) | 0.279 |
| Operation time, min | 1.001 (0.999-1.004) | 0.321 |  |  | 1.004 (1.000-1.007) | 0.060 |  |  |
| Blood transfusion | 2.684 (1.382-5.212) | **0.004** | 2.437 (0.945-6.289) | 0.065 | 2.705 (1.059-6.907) | **0.037** | 1.756 (0.155-19.913) | 0.650 |
| Anatomic resection | 0.658 (0.402-1.077) | 0.096 |  |  | 0.841 (0.432-1.639) | 0.611 |  |  |
| Single tumor | 0.676 (0.312-1.465) | 0.321 |  |  | 0.512 (0.201-1.300) | 0.159 |  |  |
| Tumor size, cm, > 5 vs. ≤ 5 | 2.142 (1.400-3.278) | **< 0.001** | 1.687 (0.953-2.986) | **0.073** | 2.062 (1.125-3.782) | **0.019** | 3.641 (1.491-8.890) | **0.005** |
| MVI | 2.145 (1.380-3.334) | **< 0.001** | 1.353 (0.741-2.469) | 0.325 | 3.087 (1.694-5.628) | **< 0.001** | 3.004 (1.269-7.108) | **0.012** |
| Satellite nodule | 3.937 (2.378-6.516) | **< 0.001** | 2.817 (1.451-5.469) | **0.002** | 1.779 (0.875-3.615) | 0.111 |  |  |
| PVTT | 4.147 (1.791-9.598) | **< 0.001** | 0.735 (0.194-2.784) | 0.650 | 7.076 (2.954-16.949) | **< 0.001** | 0.677 (0.189-2.425) | 0.549 |
| Cirrhosis | 0.809 (0.511-1.281) | 0.366 |  |  | 0.523 (0.257-1.063) | 0.073 |  |  |
| Edmondson-Steiner grade, ≥ Ⅲ vs. ≤ Ⅱ | 1.968 (1.289-3.006) | **0.002** | 0.894 (0.493-1.622) | 0.713 | 3.651 (1.949-6.838) | **< 0.001** | 5.567 (2.224-13.933) | **< 0.001** |
| BCLC stage, 0/A vs. B/C | 0.274 (0.156-0.483) | **< 0.001** | 0.211 (0.089-0.499) | **< 0.001** | 0.194 (0.101-0.374) | **< 0.001** | 0.139 (0.038-0.509) | **0.003** |

^*^ The HR (95% CI) and the corresponding p value were not available due to the extremely small number of patients with Child‒Pugh B.

Bold text indicated that these variables were statistically significant.

Abbreviations: RFS, recurrence-free survival; OS, overall survival; HBsAg, hepatitis B surface antigen; HCC, hepatocellular carcinoma; PSM, propensity score matching; HR, hazard ratio; CI, confidence interval; BMI, body mass index; ETV, entecavir; NAs, nucleos(t)ide analogues; HBsAb, hepatitis B surface antibody; HBeAb, hepatitis B e antibody; HBV, hepatitis B virus; AFP, alpha-fetoprotein; ALT, alanine aminotransferase; AST, aspartate aminotransferase; TBIL, total bilirubin; PT, prothrombin time; ASA, American Society of Anesthesiologists; MVI, microvascular invasion; PVTT, portal vein tumor thrombus; BCLC, Barcelona Clinic Liver Cancer
