# Supplementary material for: Effect of nucleos(t)ide analogue discontinuation on the prognosis of HBeAg‐negative hepatitis B virus‐related hepatocellular carcinoma after hepatectomy: A propensity score matching analysis
Source: Cancer Med. 2024 Sep 1;13(16):e70185. doi: 10.1002/cam4.70185 (PMC11366777; doi:10.1002/cam4.70185)
Supplement: Supplementary file 6 — Table S4. [file CAM4-13-e70185-s008.docx]

**Table S4. Univariate and multivariate Cox regression analysis of recurrence-free survival (RFS) and overall survival (OS) in hepatitis B surface antigen (HBsAg)-positive hepatocellular carcinoma (HCC) patients after propensity score matching (PSM)**

| **Variables** | **RFS** | | | | **OS** | | | |
| --- | --- | --- | --- | --- | --- | --- | --- | --- |
|  | **Univariate** | | **Multivariate** | | **Univariate** | | **Multivariate** | |
|  | **HR (95% CI)** | **P value** | **HR (95% CI)** | **P value** | **HR (95% CI)** | **P value** | **HR (95% CI)** | **P value** |
| Age, years | 0.979 (0.970-0.989) | **< 0.001** | 0.980 (0.969-0.990) | **< 0.001** | 0.982 (0.972-0.992) | **< 0.001** | 0.984 (0.972-0.997) | **0.017** |
| Male sex | 1.143 (0.825-1.584) | 0.421 |  |  | 1.147 (0.806-1.630) | 0.446 |  |  |
| BMI, kg/m^2^ | 0.988 (0.954-1.023) | 0.485 |  |  | 0.969 (0.933-1.007) | 0.111 |  |  |
| Alcohol consumption | 0.905 (0.719-1.138) | 0.394 |  |  | 0.882 (0.687-1.131) | 0.321 |  |  |
| Cigarette smoking | 0.962 (0.769-1.204) | 0.736 |  |  | 1.046 (0.822-1.332) | 0.712 |  |  |
| Diabetes mellitus | 0.775 (0.481-1.247) | 0.293 |  |  | 0.789 (0.476-1.309) | 0.359 |  |  |
| Hypertension | 0.760 (0.539-1.072) | 0.118 |  |  | 0.737 (0.501-1.084) | 0.121 |  |  |
| ETV monotherapy | 1.143 (0.861-1.518) | 0.356 |  |  | 1.208 (0.881-1.655) | 0.241 |  |  |
| NAs, continuation vs. discontinuation | 0.462 (0.368-0.580) | **< 0.001** | 0.400 (0.315-0.506) | **< 0.001** | 0.252 (0.193-0.328) | **< 0.001** | 0.218 (0.166-0.287) | **< 0.001** |
| HBsAb-positive | 1.097 (0.717-1.678) | 0.670 |  |  | 1.242 (0.787-1.960) | 0.352 |  |  |
| HBeAb-positive | 1.522 (0.906-2.557) | 0.113 |  |  | 2.842 (1.341-6.022) | **0.006** | 1.396 (0.644-3.030) | 0.398 |
| HBV DNA, IU/mL, > 10^3^ vs. ≤ 10^3^ | 1.394 (1.113-1.747) | **0.004** | 1.065 (0.838-1.352) | 0.608 | 1.641 (1.283-2.099) | **< 0.001** | 1.150 (0.881-1.502) | 0.304 |
| AFP, ng/mL, > 400 vs. ≤ 400 | 1.845 (1.474-2.310) | **< 0.001** | 1.395 (1.094-1.779) | **0.007** | 1.748 (1.372-2.226) | **< 0.001** | 1.332 (1.029-1.723) | **0.029** |
| Hemoglobin, g/L | 0.993 (0.987-0.999) | **0.032** | 0.995 (0.988-1.002) | 0.168 | 0.995 (0.988-1.002) | 0.163 |  |  |
| Platelets, 10^9^/L | 1.002 (1.001-1.003) | **0.007** | 0.999 (0.998-1.000) | 0.184 | 1.002 (1.000-1.003) | **0.031** | 0.999 (0.997-1.000) | 0.138 |
| ALT, IU/L | 1.001 (0.999-1.002) | 0.458 |  |  | 1.000 (0.999-1.002) | 0.719 |  |  |
| AST, IU/L | 1.001 (1.000-1.002) | 0.078 |  |  | 1.001 (1.000-1.002) | 0.124 |  |  |
| TBIL, μmol/L | 0.994 (0.978-1.011) | 0.484 |  |  | 0.998 (0.981-1.015) | 0.788 |  |  |
| Albumin, g/L | 0.974 (0.949-0.999) | **0.044** | 0.983 (0.955-1.012) | 0.239 | 0.967 (0.941-0.994) | **0.016** | 0.980 (0.951-1.010) | 0.195 |
| PT, s | 1.106 (0.986-1.241) | 0.086 |  |  | 1.199 (1.062-1.353) | **0.003** | 1.079 (0.949-1.226) | 0.247 |
| Child‒Pugh grade, A vs. B | 1.355 (0.337-5.444) | 0.669 |  |  | 2.336 (0.328-16.649) | 0.397 |  |  |
| ASA grade, Ⅱ vs. Ⅰ | 0.873 (0.685-1.113) | 0.272 |  |  | 0.761 (0.581-0.997) | **0.047** | 1.100 (0.805-1.503) | 0.548 |
| Blood loss, mL | 1.000 (1.000-1.001) | **0.009** | 1.000 (0.999-1.000) | 0.192 | 1.000 (1.000-1.001) | **0.002** | 1.000 (0.999-1.000) | 0.346 |
| Operation time, min | 1.003 (1.001-1.004) | **< 0.001** | 1.001 (1.000-1.003) | 0.099 | 1.003 (1.001-1.004) | **< 0.001** | 1.001 (1.000-1.003) | 0.157 |
| Blood transfusion | 1.275 (0.853-1.907) | 0.236 |  |  | 1.536 (1.031-2.288) | **0.035** | 1.215 (0.673-2.197) | 0.518 |
| Anatomic resection | 0.976 (0.777-1.228) | 0.838 |  |  | 1.052 (0.823-1.345) | 0.685 |  |  |
| Single tumor | 0.724 (0.557-0.941) | **0.016** | 0.639 (0.391-1.045) | 0.075 | 0.764 (0.574-1.016) | 0.064 |  |  |
| Tumor size, cm, > 5 vs. ≤ 5 | 2.568 (1.989-3.315) | **< 0.001** | 2.161 (1.623-2.877) | **< 0.001** | 2.170 (1.654-2.847) | **< 0.001** | 1.689 (1.255-2.273) | **< 0.001** |
| MVI | 2.025 (1.611-2.545) | **< 0.001** | 1.385 (1.075-1.784) | **0.012** | 2.215 (1.733-2.831) | **< 0.001** | 1.549 (1.169-2.053) | **0.002** |
| Satellite nodule | 2.078 (1.522-2.835) | **< 0.001** | 1.323 (0.937-1.867) | 0.111 | 1.894 (1.368-2.623) | **< 0.001** | 1.233 (0.844-1.802) | 0.279 |
| PVTT | 2.240 (1.669-3.006) | **< 0.001** | 1.558 (0.873-2.779) | 0.134 | 2.320 (1.714-3.140) | **< 0.001** | 0.944 (0.612-1.454) | 0.792 |
| Cirrhosis | 0.940 (0.751-1.176) | 0.587 |  |  | 1.070 (0.840-1.363) | 0.584 |  |  |
| Edmondson-Steiner grade, ≥ Ⅲ vs. ≤ Ⅱ | 1.496 (1.193-1.876) | **< 0.001** | 1.330 (1.044-1.693) | **0.021** | 1.753 (1.368-2.245) | **< 0.001** | 1.681 (1.294-2.184) | **< 0.001** |
| BCLC stage, 0/A vs. B/C | 0.475 (0.375-0.601) | **< 0.001** | 1.003 (0.572-1.761) | 0.990 | 0.439 (0.342-0.563) | **< 0.001** | 0.525 (0.375-0.734) | **< 0.001** |

Bold text indicated that these variables were statistically significant.

Abbreviations: RFS, recurrence-free survival; OS, overall survival; HBsAg, hepatitis B surface antigen; HCC, hepatocellular carcinoma; PSM, propensity score matching; HR, hazard ratio; CI, confidence interval; BMI, body mass index; ETV, entecavir; NAs, nucleos(t)ide analogues; HBsAb, hepatitis B surface antibody; HBeAb, hepatitis B e antibody; HBV, hepatitis B virus; AFP, alpha-fetoprotein; ALT, alanine aminotransferase; AST, aspartate aminotransferase; TBIL, total bilirubin; PT, prothrombin time; ASA, American Society of Anesthesiologists; MVI, microvascular invasion; PVTT, portal vein tumor thrombus; BCLC, Barcelona Clinic Liver Cancer
