# Supplementary material for: Effect of nucleos(t)ide analogue discontinuation on the prognosis of HBeAg‐negative hepatitis B virus‐related hepatocellular carcinoma after hepatectomy: A propensity score matching analysis
Source: Cancer Med. 2024 Sep 1;13(16):e70185. doi: 10.1002/cam4.70185 (PMC11366777; doi:10.1002/cam4.70185)
Supplement: Supplementary file 7 — Table S5. [file CAM4-13-e70185-s003.docx]

**Table S5. Recurrence-free survival (RFS) and overall survival (OS) of hepatocellular carcinoma (HCC) patients stratified by hepatitis B surface antigen (HBsAg) status and/or nucleos(t)ide analogue (NA) therapy before and after propensity score matching (PSM)**

| **Before PSM** | | | | | | | | | | | |
| --- | --- | --- | --- | --- | --- | --- | --- | --- | --- | --- | --- |
|  | | **RFS** | | | | | **OS** | | | | |
| **Stratification** | | **Median time, months** | **1-year rate** | **3-year rate** | **5-year rate** | **P value** | **Median time, months** | **1-year rate** | **3-year rate** | **5-year rate** | **P value** |
| Overall | Continuation of NAs | 42.4 | 72.2% | 52.5% | 44.4% | **< 0.001** | N/A^*^ | 93.9% | 79.8% | 69.7% | **< 0.001** |
|  | Discontinuation of NAs | 12.0 | 50.0% | 34.5% | 30.0% |  | 26.8 | 69.2% | 43.5% | 35.9% |  |
| HBsAg-negative | Continuation of NAs | 21.2 | 61.5% | 43.6% | 36.6% | **0.029** | N/A^*^ | 92.3% | 75.4% | 66.8% | 0.935 |
|  | Discontinuation of NAs | 60.0 | 72.1% | 55.7% | 48.2% |  | N/A^*^ | 90.0% | 75.2% | 69.0% |  |
| HBsAg-positive | Continuation of NAs | 43.8 | 73.7% | 53.8% | 45.5% | **< 0.001** | N/A^*^ | 94.2% | 80.4% | 70.1% | **< 0.001** |
|  | Discontinuation of NAs | 7.0 | 35.8% | 20.6% | 17.9% |  | 14.1 | 56.4% | 24.4% | 18.0% |  |
| **After PSM** | | | | | | | | | | | |
|  | | **RFS** | | | | | **OS** | | | | |
| **Stratification** | | **Median time, months** | **1-year rate** | **3-year rate** | **5-year rate** | **P value** | **Median time, months** | **1-year rate** | **3-year rate** | **5-year rate** | **P value** |
| Overall | Continuation of NAs | 24.6 | 60.3% | 43.5% | 38.0% | **< 0.001** | N/A^*^ | 89.8% | 71.2% | 61.2% | **< 0.001** |
|  | Discontinuation of NAs | 11.6 | 48.7% | 32.8% | 29.9% |  | 24.3 | 67.4% | 39.9% | 33.4% |  |
| HBsAg-negative | Continuation of NAs | 14.4 | 53.2% | 34.3% | 29.0% | **< 0.001** | N/A^*^ | 89.9% | 72.6% | 62.7% | 0.115 |
|  | Discontinuation of NAs | N/A^*^ | 79.6% | 62.1% | 60.2% |  | N/A^*^ | 94.3% | 81.0% | 76.7% |  |
| HBsAg-positive | Continuation of NAs | 28.4 | 62.6% | 46.4% | 40.8% | **< 0.001** | N/A^*^ | 89.7% | 70.7% | 60.6% | **< 0.001** |
|  | Discontinuation of NAs | 7.1 | 36.1% | 20.9% | 18.0% |  | 14.2 | 57.3% | 24.9% | 18.9% |  |

^*^The corresponding median survival times were not reached.

Bold text indicated that these variables were statistically significant.

Abbreviations: RFS, recurrence-free survival; OS, overall survival; HCC, hepatocellular carcinoma; HBsAg, hepatitis B surface antigen; NAs, nucleos(t)ide analogues; PSM, propensity score matching; N/A, not applicable
