# Supplementary material for: Effect of nucleos(t)ide analogue discontinuation on the prognosis of HBeAg‐negative hepatitis B virus‐related hepatocellular carcinoma after hepatectomy: A propensity score matching analysis
Source: Cancer Med. 2024 Sep 1;13(16):e70185. doi: 10.1002/cam4.70185 (PMC11366777; doi:10.1002/cam4.70185)
Supplement: Supplementary file 8 — Table S6. [file CAM4-13-e70185-s001.docx]

**Table S6. Baseline characteristics of hepatitis B surface antigen (HBsAg)-negative hepatocellular carcinoma (HCC) patients before and after propensity score matching (PSM)**

| **Variables** | **Before PSM** | | | **After PSM** | | |
| --- | --- | --- | --- | --- | --- | --- |
|  | **Continuation of NAs (N=104)** | **Discontinuation of NAs (N=150)** | **P value** | **Continuation of NAs (N=70)** | **Discontinuation of NAs (N=70)** | **P value** |
| **Demographic characteristics** |  |  |  |  |  |  |
| Age, years | 58.9 ± 10.3 | 59.9 ± 10.6 | 0.430 | 58.3 ± 10.2 | 58.9 ± 10.8 | 0.627 |
| Male sex | 97 (93.3%) | 137 (91.3%) | 0.573 | 67 (95.7%) | 65 (92.9%) | 0.718 |
| BMI, kg/m^2^ | 22.7 ± 3.1 | 23.6 ± 3.4 | 0.103 | 23.0 ± 3.1 | 23.5 ± 3.4 | 0.934 |
| Alcohol consumption | 44 (42.3%) | 76 (50.7%) | 0.189 | 30 (42.9%) | 35 (50.0%) | 0.397 |
| Cigarette smoking | 60 (57.7%) | 89 (59.3%) | 0.794 | 42 (60.0%) | 48 (68.6%) | 0.290 |
| Diabetes mellitus | 12 (11.5%) | 19 (12.7%) | 0.787 | 8 (11.4%) | 13 (18.6%) | 0.237 |
| Hypertension | 40 (38.5%) | 35 (23.3%) | **0.009** | 24 (34.3%) | 20 (28.6%) | 0.466 |
| Type of NA |  |  | 0.440 |  |  | 0.547 |
| ETV | 78 (75.0%) | 124 (82.7%) |  | 54 (77.1%) | 60 (85.7%) |  |
| TDF | 5 (4.8%) | 5 (3.3%) |  | 2 (2.9%) | 2 (2.9%) |  |
| Other | 5 (4.8%) | 7 (4.7%) |  | 4 (5.7%) | 3 (4.3%) |  |
| Exposure to two or more types of NAs | 16 (15.4%) | 14 (9.3%) |  | 10 (14.3%) | 5 (7.1%) |  |
| **Laboratory findings** |  |  |  |  |  |  |
| HBsAb-positive | 76 (73.1%) | 98 (65.3%) | 0.191 | 50 (71.4%) | 50 (71.4%) | 1.000 |
| HBeAb-positive | 68 (65.4%) | 102 (68.0%) | 0.663 | 54 (77.1%) | 48 (68.6%) | 0.254 |
| HBV DNA, IU/mL |  |  | **< 0.001** |  |  | 0.288 |
| ≤ 10^3^ | 80 (76.9%) | 142 (94.7%) |  | 60 (85.7%) | 64 (91.4%) |  |
| > 10^3^ | 24 (23.1%) | 8 (5.3%) |  | 10 (14.3%) | 6 (8.6%) |  |
| AFP, ng/mL |  |  | 0.919 |  |  | 0.716 |
| ≤ 400 | 68 (65.4%) | 99 (66.0%) |  | 47 (67.1%) | 49 (70.0%) |  |
| > 400 | 36 (34.6%) | 51 (34.0%) |  | 23 (32.9%) | 21 (30.0%) |  |
| Hemoglobin, g/L | 143.5 [129.0-152.0] | 142.0 [129.0-151.0] | 0.559 | 144.0 [134.0-152.0] | 140.5 [132.2-153.0] | 0.611 |
| Platelets, 10^9^/L | 163.5 [118.0-203.0] | 150.5 [121.5-201.8] | 0.879 | 167.0 [135.2-217.0] | 163.0 [129.0-203.2] | 0.683 |
| ALT, IU/L | 30.0 [24.0-37.0] | 27.0 [20.0-42.0] | 0.700 | 29.5 [24.0-39.0] | 26.0 [19.2-38.5] | 0.214 |
| AST, IU/L | 32.5 [24.0-45.0] | 30.0 [24.0-48.0] | 0.819 | 27.0 [21.0-42.0] | 29.5 [24.0-38.0] | 0.980 |
| TBIL, μmol/L | 12.0 [10.0-15.8] | 14.0 [10.2-18.2] | **0.022** | 12.7 [10.4-17.4] | 13.2 [9.6-17.3] | 0.882 |
| Albumin, g/L | 42.9 [40.4-45.2] | 43.4 [40.8-46.2] | 0.341 | 43.8 [40.1-45.7] | 43.5 [41.3-46.2] | 0.480 |
| PT, s | 11.4 [11.1-12.1] | 11.6 [11.0-12.3] | 0.843 | 11.5 [11.1-12.1] | 11.5 [11.0-12.4] | 0.407 |
| Child‒Pugh grade |  |  | 0.162 |  |  | 0.366 |
| A | 100 (96.2%) | 149 (99.3%) |  | 66 (94.3%) | 69 (98.6%) |  |
| B | 4 (3.8%) | 1 (0.7%) |  | 4 (5.7%) | 1 (1.4%) |  |
| **Surgical data** |  |  |  |  |  |  |
| ASA grade |  |  | 0.560 |  |  | 0.307 |
| Ⅰ | 44 (42.3%) | 69 (46.0%) |  | 34 (48.6%) | 28 (40.0%) |  |
| Ⅱ | 60 (57.7%) | 81 (54.0%) |  | 36 (51.4%) | 42 (60.0%) |  |
| Blood loss, mL | 300.0 [200.0-400.0] | 300.0 [150.0-400.0] | 0.067 | 300.0 [200.0-400.0] | 300.0 [200.0-400.0] | 0.162 |
| Operation time, min | 215.0 [175.0-260.0] | 200.0 [160.0-244.0] | **0.033** | 210.0 [175.0-257.5] | 200.0 [163.8-235.0] | 0.197 |
| Blood transfusion | 12 (11.5%) | 6 (4.0%) | **0.021** | 6 (8.6%) | 5 (7.1%) | 0.753 |
| Anatomic resection | 24 (23.1%) | 61 (40.7%) | **0.003** | 23 (32.9%) | 23 (32.9%) | 1.000 |
| **Pathologic findings** |  |  |  |  |  |  |
| Single tumor | 101 (97.1%) | 130 (86.7%) | **0.004** | 67 (95.7%) | 67 (95.7%) | 1.000 |
| Tumor size, cm |  |  | **0.032** |  |  | 0.862 |
| ≤ 5 | 60 (57.7%) | 66 (44.0%) |  | 44 (62.9%) | 43 (61.4%) |  |
| > 5 | 44 (42.3%) | 84 (56.0%) |  | 26 (37.1%) | 27 (38.6%) |  |
| MVI | 32 (30.8%) | 45 (30.0%) | 0.896 | 18 (25.7%) | 17 (24.3%) | 0.845 |
| Satellite nodule | 16 (15.4%) | 15 (10.0%) | 0.197 | 14 (20.0%) | 7 (10.0%) | 0.098 |
| PVTT | 6 (5.8%) | 9 (6.0%) | 0.939 | 5 (7.1%) | 8 (11.4%) | 0.382 |
| Cirrhosis | 49 (47.1%) | 31 (20.7%) | **< 0.001** | 28 (40.0%) | 25 (35.7%) | 0.601 |
| Edmondson-Steiner grade |  |  | 0.051 |  |  | 0.229 |
| ≤ Ⅱ | 56 (53.8%) | 99 (66.0%) |  | 38 (54.3%) | 45 (64.3%) |  |
| ≥ Ⅲ | 48 (46.2%) | 51 (34.0%) |  | 32 (45.7%) | 25 (35.7%) |  |
| BCLC stage |  |  | 0.316 |  |  | 0.629 |
| 0/A | 92 (88.5%) | 126 (84.0%) |  | 59 (84.3%) | 61 (87.1%) |  |
| B/C | 12 (11.5%) | 24 (16.0%) |  | 11 (15.7%) | 9 (12.9%) |  |

Bold text indicated that these variables were statistically significant.

Abbreviations: HBeAg, hepatitis B virus e antigen; HBV, hepatitis B virus; HCC, hepatocellular carcinoma; PSM, propensity score matching; NAs, nucleos(t)ide analogues; BMI, body mass index; ETV, entecavir; TDF, tenofovir disoproxil fumarate; HBsAg, hepatitis B surface antigen; HBsAb, hepatitis B surface antibody; HBeAb, hepatitis B e antibody; AFP, alpha-fetoprotein; ALT, alanine aminotransferase; AST, aspartate aminotransferase; TBIL, total bilirubin; PT, prothrombin time; ASA, American Society of Anesthesiologists; MVI, microvascular invasion; PVTT, portal vein tumor thrombus; BCLC, Barcelona Clinic Liver Cancer
